# Multi-Trait Genomic Prediction Models Enhance the Predictive Ability of Grain Trace Elements in Rice

**DOI:** 10.3389/fgene.2022.883853

**Published:** 2022-06-22

**Authors:** Blaise Pascal Muvunyi, Wenli Zou, Junhui Zhan, Sang He, Guoyou Ye

**Affiliations:** ^1^ CAAS-IRRI Joint Laboratory for Genomics-Assisted Germplasm Enhancement, Agricultural Genomics Institute in Shenzhen, Chinese Academy of Agricultural Sciences, Shenzhen, China; ^2^ Rice Breeding Innovations Platform, International Rice Research Institute, Los Baños, Philippines

**Keywords:** rice, trace elements, multi-trait genomic prediction, local epistatic effect, seletive marker panel

## Abstract

Multi-trait (MT) genomic prediction models enable breeders to save phenotyping resources and increase the prediction accuracy of unobserved target traits by exploiting available information from non-target or auxiliary traits. Our study evaluated different MT models using 250 rice accessions from Asian countries genotyped and phenotyped for grain content of zinc (Zn), iron (Fe), copper (Cu), manganese (Mn), and cadmium (Cd). The predictive performance of MT models compared to a traditional single trait (ST) model was assessed by 1) applying different cross-validation strategies (CV1, CV2, and CV3) inferring varied phenotyping patterns and budgets; 2) accounting for local epistatic effects along with the main additive effect in MT models; and 3) using a selective marker panel composed of trait-associated SNPs in MT models. MT models were not statistically significantly (*p <* 0.05) superior to ST model under CV1, where no phenotypic information was available for the accessions in the test set. After including phenotypes from auxiliary traits in both training and test sets (MT-CV2) or simply in the test set (MT-CV3), MT models significantly (*p* < 0.05) outperformed ST model for all the traits. The highest increases in the predictive ability of MT models relative to ST models were 11.1% (Mn), 11.5 (Cd), 33.3% (Fe), 95.2% (Cu) and 126% (Zn). Accounting for the local epistatic effects using a haplotype-based model further improved the predictive ability of MT models by 4.6% (Cu), 3.8% (Zn), and 3.5% (Cd) relative to MT models with only additive effects. The predictive ability of the haplotype-based model was not improved after optimizing the marker panel by only considering the markers associated with the traits. This study first assessed the local epistatic effects and marker optimization strategies in the MT genomic prediction framework and then illustrated the power of the MT model in predicting trace element traits in rice for the effective use of genetic resources to improve the nutritional quality of rice grain.

## Introduction

Over half of the world’s population relies on rice as a staple crop (Bandumula, 2018). Growing and consuming rice has relative merits, as rice is the major dietary source for both toxic and essential trace elements (Yang et al., 2018). For instance, Cd is a potent environmental and human health toxicant (Arao and Ae, 2003; Uraguchi and Fujiwara, 2012; Lien et al., 2021) transported into rice grain *via* the same channels with other trace elements Zn, Fe, Cu, and Mn (Sasaki et al., 2012; Hao et al., 2018; Han et al., 2021) of essential nutritional and physiological functions to plants, animal and humans species (Miller, 1970; Olivares and Uauy, 1996; White and Broadley, 2009; Aschner and Erikson, 2017; Gao and Xiong, 2018).

Recent advancements in genomic research and the increasing number of germplasm resources in gene banks offer a great opportunity to develop safe and nutritious rice varieties cost-effectively. The trait’s heritability indicates the potential that a given trait can be genetically improved. Previously, broad sense heritability of grain Zn, Fe, Cu, Mn, and Cd was found to be low (0–0.3), moderate (0.4–0.6) to high (0.6 or higher) (Norton et al., 2010; Pinson et al., 2015; Naik et al., 2020), indicating the practical possibility to improve these traits via selective breeding methods. Furthermore, many molecular genetic studies have identified numerous quantitative trait loci (QTLs) responsible for trace element uptake, transport, and accumulation into different rice tissues through genome-wide association studies (GWAS) or QTL mapping (Lu et al., 2008; Garcia-Oliveira et al., 2009; Ueno et al., 2010; Du et al., 2013; Huang et al., 2016; Meng et al., 2017; Swamy et al., 2018; Yang et al., 2018; Descalsota-Empleo et al., 2019; Frouin et al., 2019; Liu J. et al., 2021). As a genomics-enabled breeding approach, marker-assisted selection (MAS) is useful to improve trace element traits when genes/QTLs with large additive genetic effects exist (Wu et al., 2020). However, prominent non-additive gene action has also been reported for trace element traits, making MAS-based strategies ineffective (Sharma V. et al., 2021). In addition, MAS-based breeding methods are practically ineffective at simultaneously exploiting information from multiple genes (Spindel et al., 2016) or traits (Van Der Straeten et al., 2020).

In contrast, genomic selection (GS) approaches make use of total genome-wide markers with either large additive effects or minor effects to derive the genomic estimated genetic values of genotypes (Meuwissen et al., 2001), which overcomes the constraints of MAS-based methods (de los Campos et al., 2009). Also, GS models can be modified to a multi-trait (MT) form to exploit available information from multiple traits simultaneously. The MT models used in GS heavily rely on genetic correlation between traits (Henderson and Quaas, 1976). This correlation possibly results from the pleiotropic effect (multiple traits controlled by the same QTL) or linkage disequilibrium (LD) between genes (Falconer, 1996). Exploiting multi-trait information in GS has been awarded with an increase in prediction accuracy ranging from 24% to 105% relative to single trait (ST) models (Rutkoski et al., 2016; Sun et al., 2017; Arojju et al., 2020). Besides gains in prediction accuracy, integrating MT models with appropriate cross-validation (CV) schemes compensated for the negative effect of small population size without affecting the prediction accuracy, enabling breeders to minimize phenotyping budgets (Lado et al., 2018; Arojju et al., 2020). The benefits of MT models under various CV schemes are yet to be studied in diverse rice collections. Nevertheless, MT models have shown their potential in predicting complex traits in rice, such as grain arsenic content (Ahmadi et al., 2021), grain yield (Wang et al., 2017), and root index architecture (Sharma S. et al., 2021).

Most of the MT genomic prediction studies discussed above only modeled the additive genetic effects. Non-additive effects are also essential components of the genetic effect and can benefit the predictive ability of MT models if accommodated (dos Santos et al., 2016; Lyra et al., 2017). However, non-additive effects such as dominance or global epistatic effects may not be conserved during breeding due to chromosomal recombination events (Falconer, 1996; Breseghello and Sorrells, 2006; He et al., 2017). In contrast, the local epistasis that spans short segments of chromosomes can be preserved over generations (Akdemir and Jannink, 2015), as adjacent loci normally hold a strong LD (Ardlie et al., 2002). Earlier GS studies with ST models illustrated that accounting for local epistatic effects along with the main additive model increased the prediction accuracy of agronomic traits in wheat accessions (Akdemir and Jannink, 2015; Akdemir et al., 2017; He et al., 2017; Jiang et al., 2018; He et al., 2019). However, the benefits of modeling local epistasis effects in MT models remain unknown in crop or animal species.

Genomic prediction models can be extended to incorporate markers associated with causal QTLs, such as trait-associated SNPs (TA-SNPs), bridging the gap between biology and mechanistic GS models using uninformative genome-wide markers. Also, genomic prediction with markers derived from functional QTL is less reliant on LD patterns shared by training and target populations, possibly allowing robust prediction, especially across unrelated populations where LD decays more rapidly (Snelling et al., 2013). Simulation and empirical studies have shown that accounting for known QTLs improves the performance of genomic prediction models compared to models using uninformative genome-wide markers (Bernardo, 2014; Owens et al., 2014). Alemu et al. (2021); Zhou et al. (2021) reported a two- to four-fold gain in prediction accuracy using GS + *de novo* GWAS (Spindel et al., 2016), in which the most significant TA-SNPs from a GWAS conducted on the training population are fitted as fixed effects in the model along with the polygenic background. Other groups (Bhandari et al., 2019; Ahmadi et al., 2021) also reported gains in prediction accuracy ranging from 16% to 32% by exploiting GWAS-derived TA-SNPs using trait-specific genomic relationship matrices (Zhang et al., 2014) in which markers with stronger association signals are assigned higher weights than markers with weaker associations. However, the application of the above methods has not always been beneficial (Veerkamp et al., 2016; Rice and Lipka, 2019) and has been shown to depend on the genetic architecture of the traits of interest, trait heritability, the number of underlying causal mutations, and their effect sizes (Huang and Mackay, 2016). In addition, the use of TA-SNPs in genomic prediction has been scarcely investigated in models accounting for the non-additive effects. The potential of GWAS-derived TA-SNPs on the predictive ability of MT models accounting for the local epistatic effects in diverse rice populations is yet to be demonstrated and worth inspecting.

There is a great scope for applying MT models to evaluate trace elements in large germplasm collections such as those archived in gene banks. Earlier studies using ST models showed that GS is a robust and cost-efficient tool to predict the genetic merit of individuals in large germplasm collections for various agronomic traits, such as grain yield in rice (Tanaka et al., 2021), biomass yield in sorghum (Yu et al., 2016), oil, protein, and yield in soybean (Jarquin et al., 2016), total root length in maize (Pace et al., 2015), and days to head and days to maturity in wheat (Crossa et al., 2017). However, the application of MT or even ST models to predict the concentrations of trace elements in food crops is still limited to a few studies involving arsenic (Frouin et al., 2019; Ahmadi et al., 2021), Mn (Leplat et al., 2016), and Zn (Guo et al., 2020) in rice, barley, and maize grain, respectively. Therefore, the overall goal of the present study is to compare the robustness of ST and MT models in predicting concentrations of four essential trace elements, Fe, Zn, Cu, and Mn, and one toxic metal, Cd, in rice grain. Different CV schemes, implying varied phenotyping patterns and costs, were examined in our study to seek the most efficient phenotyping strategy when multiple traits are planned to be measured. In addition, we investigated whether incorporating local epistatic effects and using a selective marker panel of TA-SNPs derived from GWAS into MT models could further enhance the predictive ability of MT models.

## Materials and Methods

### Rice Materials

Our study used 250 rice accessions, including *indica* and *japonica* ecotype accessions from Asian countries (Supplementary Table S1). Accessions from China are mainly landrace *indica* varieties mostly cultivated on Cd-polluted soils in Guangdong province, China (Long et al., 2014).

### Plant Cultivation and Quantification of Trace Elements in Rice Grain

The procedures followed for growing the 250 accessions and determining concentrations of trace elements in rice grain were as previously described by Liu S. et al. (2021). Briefly, seeds from the 250 accessions were first cultivated in pots filled with soil collected from the experimental station of the Agricultural Genomics Institute at Shenzhen, China. Next, germinated seeds were selected and cultivated in seedling trays for 4 weeks. Healthy seedlings were then transferred into pots containing soil amended with an initial concentration of Cd of 0.5 mg kg^−1^. Finally, all the seedlings were planted in an augmented randomized complete block design with two replicates of 25 accessions from 20 July 2019 to 2 October 2019. Our study was limited to a single environment. Multi-environment data would be essential for understanding the environmental correlations and their stability and genotype effects by environment interactions (GxE). To determine grain concentrations of Zn, Fe, Cu, Mn, and Cd, grain samples were first peeled and dried at 65°C for 3 days. The dried samples were then crushed, wet-digested in concentrated nitric acid (HNO_3_) at 120°C for 30 min, and further digested with perchloric acid (HClO_4_) at 180°C until the samples became transparent. The samples were then diluted with ultrapure water. Finally, the grain concentration of each trace element was determined using the Inductively Coupled Plasma Mass Spectrometry (ICP-MS) machine (Houk et al., 1980).

### Genotyping

The 250 accessions were genotyped following the re-sequencing and variants-calling procedures of the rice 3K project as reported by Wang et al. (2018). The following steps were implemented for all the genotypes to merge the variants-calling: First, raw reads were aligned to the R498 reference genome (Du et al., 2017) using the program bwa-mem alignment software (Li, 2013). Next, the PCR duplicates were identified with Picard software, version 2.9.0 (http://broadinstitute.github.io/picard/), and discarded. Following that, the GATK HaplotypeCaller engine (McKenna et al., 2010), with the option “-ERCGVCF,” was used to call genotypes at each site. The resulting genomic variants called format (gVCF) for each genotype were combined using the GATK Genotype GVCFs engine. Next, the GATK hard filter pipeline was used to individually call SNP and INDEL variants from the population variant file. All the variants within 5 bp of an INDEL were discarded. A variant was confirmed if at least one genotype supported it with a QUAL parameter greater than 30. After that, VCF tools indicated (Danecek et al., 2011) sites for which genotypes were not called in at least 20% of the used genotypes. The above procedures yielded 30,089,814 bi-allelic SNPs for the 250 genotypes. SNP quality control steps were implemented using PLINK software (Chang et al., 2015) with standards that remove SNPs with 1) minor allele frequency lower than 0.5, 2) call rate less than 0.9, and 3) pairwise LD (*r* ^2^) greater than 0.01. Finally, 36,171 SNPs were available for the 250 accessions.

### Estimation of Genomic Heritability and Traits Genomic Correlation

The mixed linear model was used to estimate genomic heritability as follows:

$$y=1_{n}\mu +g+e$$

Where y is the vector of concentration of trace element under consideration, $1_{n}$ is the vector of ones, n is the number of genotyped cultivars, μ is the intercept, g is the vector of the genetic effects of accessions, e is the residual vector. g and e are assumed as random effects, respectively, following $g∼N\left(0,G\sigma_{g}^{2}\right)$ and $e∼N\left(0,I\sigma_{e}^{2}\right)$ where G is genomic relationship matrix estimated following (Yang et al., 2010) and I is identity matrix. The genomic heritability was estimated as

$$h_{g}^{2}=\frac{\sigma_{g}^{2}}{\left(\sigma_{g}^{2}+\sigma_{e}^{2}\right)}$$

Where $\sigma_{g}^{2}$ is the additive genetic variance component and $\sigma_{e}^{2}\;$ is the residual variance component. GCTA software (Yang et al., 2011) was used to compute the genomic relationship matrix and genomic heritability.

The genomic correlation between traits was estimated using the formula: $\mathrm{cor}=\frac{\mathrm{co}v_{i,j}}{\sigma_{i}\sigma_{j}}$ where $cov_{i,j}$ is the genetic covariance between i^th^ trait and j^th^ trait, $\sigma_{i}$ and $\sigma_{j}$ are the square root of the genetic variance of i^th^ trait and j^th^ trait. The genetic covariances and variances were estimated using the R package MTM (De los Campos and Grüneberg, 2016).

### Genetic Diversity

A hierarchical cluster analysis based on the Euclidean’s distance matrix computed with the SNP genomic profiles was performed to inspect the genetic diversity among the 250 genotypes. In addition, a heat plot based on the cluster analysis was drawn to visualize the genetic dissimilarities.

### Genomic Prediction Approaches

The genomic prediction models used were the ST and MT models. The ST model only captured additive genetic effects, while the MT models accommodated both additive and local epistatic effects. ST model was the commonly used genomic best linear unbiased prediction (GBLUP) model and same as the mixed model estimating genomic heritability: $y=1_{n}\mu +g+e$ , where y , $1_{n}$ , μ and ε were exactly as afore denoted, g is the vector of additive genetic effect in genotype-based model or additive plus local epistatic effects in the haplotype-based model. In the genotype-based model, we assumed $g∼N\left(0,G\sigma_{a}^{2}\right)$ , where G is an n × n-dimensional additive genomic relationship matrix, and $\sigma_{a}^{2}$ is the additive genetic variance component. In the haplotype-based model, we assumed $g∼N\left(0,H\sigma_{h}^{2}\right)$ , where H is an n × n-dimensional haplotypic relationship matrix derived from the haplotypic profile matrix with values 0, 1, and 2 indicating the number of copies for a specific haplotypic allele (Jiang et al., 2018; He et al., 2019). To obtain the haplotypic profile matrix, the genotypic data of SNPs were phased using software SHAPEIT (Delaneau et al., 2012) with default augment settings. The phased genotypic data was recoded to haplotypic profiles using a fixed-length haplotype of 2, 3, 4, or 5 SNPs. G and H were established using software GCTA (Yang et al., 2010; Yang et al., 2011) based on the genotypic and haplotypic data, respectively.

For MT models, we used two approaches considering no correlation between traits; the Bayesian multi-output regressor stacking (BMORS) proposed by Montesinos-López et al. (2019); the MT-GBLUP; and two methods accommodating correlation between traits; the factorial analytic (FA) model and the unstructured (UN) model. For genotype-based approaches, the MT-GBLUP model was formulated as y=μ+u+ε , where $y={\left(y_{1}^{\prime },y_{2}^{\prime },\ldots ,y_{m}^{\prime }\right)}^{\prime }$ , $\mu ={\left(\mu_{1}^{\prime },\mu_{2}^{\prime },\ldots ,\mu_{m}^{\prime }\right)}^{\prime }$ , $u={\left(g_{1}^{\prime },g_{2}^{\prime },\ldots ,g_{m}^{\prime }\right)}^{\prime }$ , $\varepsilon ={\left(e_{1}^{\prime },e_{2}^{\prime },\ldots ,e_{m}^{\prime }\right)}^{\prime }$ , m is the number of traits included in the model. We assumed $u∼N\left({\left(0^{\prime },0^{\prime },\ldots ,0^{\prime }\right)}^{\prime },\Psi_{u}⊗G\right)$ , $\varepsilon ∼N\left({\left(0^{\prime },0^{\prime },\ldots ,0^{\prime }\right)}^{\prime },\Psi_{\varepsilon }⊗I\right)$ where $\Psi_{u}=\left(\begin{array}{ccccc} \sigma_{g_{1}}^{2} & \cdots & 0 & \cdots & 0\\ \vdots & \ddots & \vdots & \ddots & \vdots \\ 0 & \cdots & \sigma_{g_{j}}^{2} & \cdots & 0\\ \vdots & \ddots & \vdots & \ddots & \vdots \\ 0 & \cdots & 0 & \cdots & \sigma_{g_{m}}^{2} \end{array}\right),$ $\Psi_{\varepsilon }=\left(\begin{array}{ccccc} \sigma_{\varepsilon_{1}}^{2} & \cdots & 0 & \cdots & 0\\ \vdots & \ddots & \vdots & \ddots & \vdots \\ 0 & \cdots & \sigma_{\varepsilon_{j}}^{2} & \cdots & 0\\ \vdots & \ddots & \vdots & \ddots & \vdots \\ 0 & \cdots & 0 & \cdots & \sigma_{\varepsilon_{m}}^{2} \end{array}\right)$ , $\sigma_{g_{j}}^{2}$ and $\sigma_{\varepsilon_{j}}^{2}$ are respectively the genetic and residual variance of *j*th trait, and ⊗ denotes Kronecker product of matrices.

BMORS was a two-stage process. The first stage is the same as the MT-GBLUP, but instead of directly using the GBLUP predicted values as the final output, BMORS implemented a second stage that integrated the GBLUP predicted values of each trait in the first stage and fitted a ridge regression model. In this way, the prediction of a single trait could be corrected by the predictions of other traits in the first stage using the second stage model (Spyromitros-Xioufis et al., 2012; Spyromitros-Xioufis et al., 2016; Montesinos-López et al., 2019). The FA model was also based on the formula of the MT-GBLUP model but assuming a covariance structure between traits, that is $\Psi_{u}=\left(\begin{array}{ccccc} \sigma_{g_{1}}^{2} & \cdots & cov_{g_{1}g_{j}} & \cdots & cov_{g_{1}g_{m}}\\ \vdots & \ddots & \vdots & \ddots & \vdots \\ cov_{g_{j}g_{1}} & \cdots & \sigma_{g_{j}}^{2} & \cdots & cov_{g_{j}g_{m}}\\ \vdots & \ddots & \vdots & \ddots & \vdots \\ cov_{g_{m}g_{1}} & \cdots & cov_{g_{m}g_{j}} & \cdots & \sigma_{g_{m}}^{2} \end{array}\right)=\left(\Lambda \Lambda^{\prime }+\Pi \right)=FA\left(k\right)$ where k is the number of latent factors, Λ is a j × k dimensional matrix containing trait loadings, Π is a j × j diagonal matrix (Burgueño et al., 2012). Theoretically, the FA model requires at least three traits to be simultaneously included in the model. The UN model (Burgueño et al., 2012; Cuevas et al., 2017) tried to estimate all variances and covariances in $\Psi_{u}$ , i.e., $\sigma_{g_{j}}^{2}$ and $cov_{g_{i}g_{j}}$ , i,j∈{1,…,m} , which may cause convergence problems when a large number of traits are considered. The haplotype-based approach was only implemented in the MT-UN model by replacing the relationship matrix G by H .

The ST-GBLUP model was implemented in R (R Core Team, 2016) using the BGLR package (De los Campos and Pérez-Rodríguez, 2015). The MT-GBLUP, FA, and UN approaches were realized using the R package MTM (De los Campos and Grüneberg 2016). BMORS was fitted using the R package BMTME (Montesinos-López et al., 2019). The number of iterations of all models was set to 20,000, and the first 12,000 were discarded as burn-in.

### Cross-Validation Schemes and Evaluation of Genomic Prediction Accuracy

Four different CV schemes, referring to those reported by Lado et al. (2018) and Arojju et al. (2020) were used in our study (Table 1). CV1 was applied to both ST-GBLUP and MT models, referring to a scenario where the target trait was predicted without the support of auxiliary traits (ST-CV1) or with auxiliary traits only available in the training set (MT-CV1). CV2 and CV3 were only assessed for MT models. Under CV2 scheme, genotypes in both training and test sets had phenotypic data for all the auxiliary traits. Under CV3, phenotypes of the auxiliary traits were only available in the test set.

**TABLE 1:** Investigated single trait (ST) and multi-trait (MT) cross-validation (CV) schemes.

	Training set		Test set	
	Target traits	Auxiliary traits	Target traits	Auxiliary traits
ST-CV1	**Zn/Cu/Fe/Cd/Mn**	—	**Zn/Cu/Fe/Cd/Mn**	**—**
MT-CV1	**Zn**	Mn, Fe, Cu, Cd	**Zn**
	**Cu**	Mn, Fe, Zn, Cd	**Cu**
	**Fe**	Mn, Cu, Zn, Cd	**Fe**
	**Cd**	Mn, Fe, Cu, Zn	**Cd**
	**Mn**	Fe, Cu, Zn, Cd	**Mn**
MT-CV2	**Zn**	Mn, Fe, Cu, Cd	**Zn**	Mn, Fe, Cu, Cd
	**Cu**	Mn, Fe, Zn, Cd	**Cu**	Mn, Fe, Zn, Cd
	**Fe**	Mn, Cu, Zn, Cd	**Fe**	Mn, Cu, Zn, Cd
	**Cd**	Mn, Fe, Cu, Zn	**Cd**	Mn, Fe, Cu, Zn
	**Mn**	Fe, Cu, Zn, Cd	**Mn**	Fe, Cu, Zn, Cd
MT-CV3	**Zn**	—	**Zn**	Mn, Fe, Cu, Cd
	**Cu**		**Cu**	Mn, Fe, Zn, Cd
	**Fe**		**Fe**	Mn, Cu, Zn, Cd
	**Cd**		**Cd**	Mn, Fe, Cu, Zn
	**Mn**		**Mn**	Fe, Cu, Zn, Cd

Phenotypes for auxiliary traits are not available The unobserved target traits to be predicted are highlighted with bold font.

To assess the genomic prediction accuracy across the above CV schemes, the entire population of 250 genotypes was randomly divided into five equal-sized folds. Four folds collectively constituted the training set, and the remaining fold was the test set. Stochastic partitioning of training and test sets was repeated 20 times, yielding one hundred times (5 folds × 20 replicates) calibrations and predictions. The genomic prediction accuracy of the target trait was estimated using the Pearson correlation coefficient between the genomic predicted genetic values and the observed phenotypic values of 250 accessions when incorporated in the five test sets of each repeat of CV. The Student *t*-test was used to test the statistical difference in genomic prediction accuracies among the prediction models.

### Selective SNP Marker Panel

To investigate whether the predictive ability of MT genomic prediction on rice grain trace elements concentration could be boosted by optimizing the SNP marker panel, we applied a (GWAS) to identify the trait-associated SNPs (TA-SNPs) and establish the selective marker panel. The CV scenarios in which the MT-UN haplotype-based models disregarding the length of haplotypes (two to five SNPs) constantly showed statistically significantly (*p* < 0.05, *t*-test) higher prediction accuracies than their genotype-based counterparts and the ST-GBLUP model were used to validate the efficacy of using the TA-SNPs to train the genomic prediction models. In more detail, GWAS using the total SNP marker panel was performed in the training set of the designated CV scenarios. First, the TA-SNPs with *p* values less than 0.01 were recorded. Then each chromosome was divided into bins spanning 300 kb (the bin size is decided by the LD decay, with the physical distance between pairs of SNPs based on the total population). Finally, the most trait-associated SNPs with the lowest *p-*value in each bin was picked together with the TA-SNPs (*p* < 0.01) and recorded to constitute the selective SNP marker panel of each repeat of CV. The MT-UN genotype-based model was implemented using the genotypic data of the selective SNPs. Contrastingly, the adjacent selective SNPs located within 300 kb (highly possible as the position of the selective SNP from each bin is unfixed) were combined to compile the haplotypes using the phased genotypic data as the LD decay implied a non-negligible LD among them. The remaining SNPs without close neighbors within 300 kb were maintained, and their genotypic profiles were used. Therefore, the MT-UN haplotype-based model took advantage of both haplotypes and genotypes. The GWAS was implemented using GCTA software (Yang et al., 2011; Yang et al., 2014). The additive genomic relationship matrix was exclusively used in the GWAS model to account for the relatedness between accessions.

## Results

### Linkage Disequilibrium Decay, Kinship, and Population Structure

**FIGURE 1:**
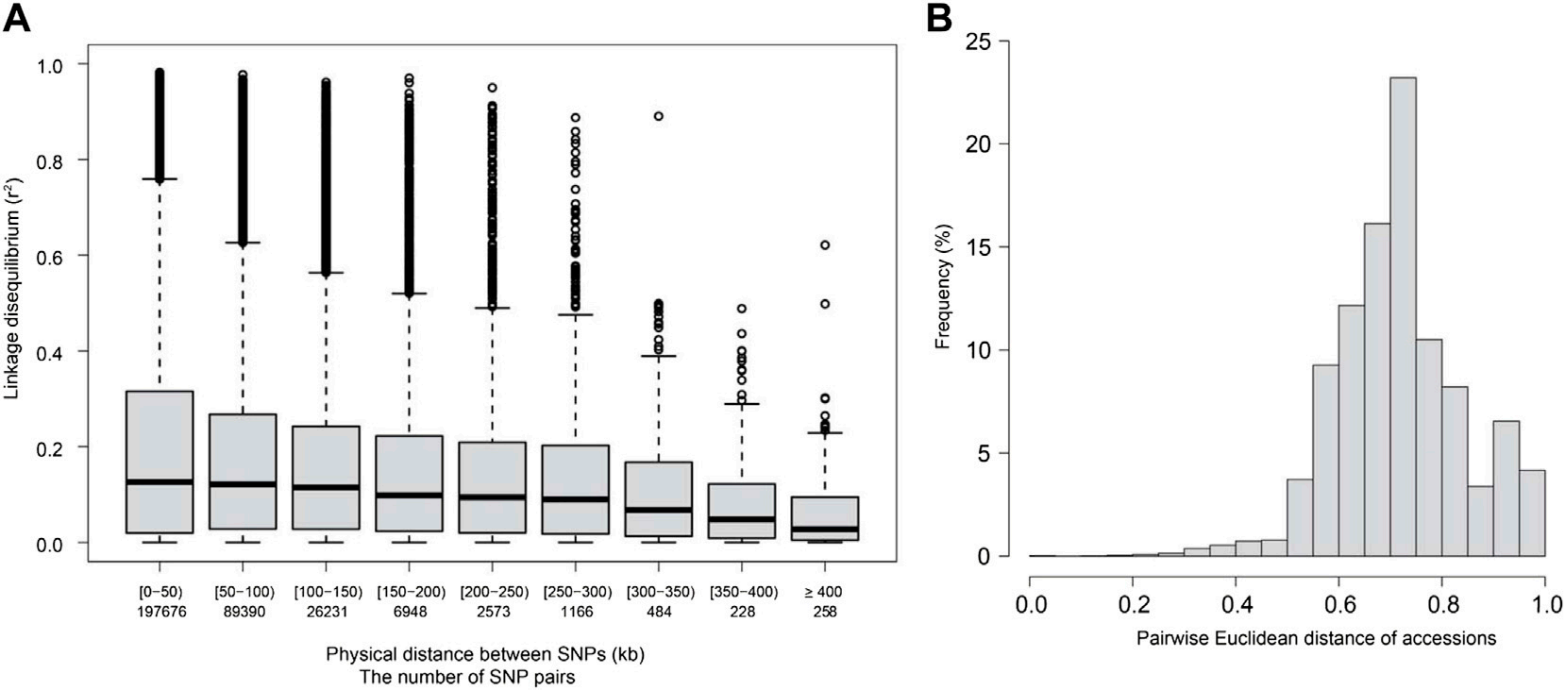
Linkage disequilibrium (LD, *r* ^2^) decay and Euclidean distance for the 250 diverse rice accessions used in this study. **(A)** LD decay for the studied accessions. The *X*-axis represents the physical distance between SNP pairs in kilobases (kb). **(B)** Pairwise Euclidean distance for the studied accessions.

The LD decay distance between all SNP markers for the 250 accessions was ∼250–300 kb when the cut-off value (*r* ^2^) was set at 0.1, assuming non-negligible SNP pairwise correlation (r = 0.3) (Figure 1A). The kinship between accessions was determined based on pairwise Euclidean distances. Pairwise distances among accessions ranging from 0 to 0.2 accounted for less than 5% of all the pairwise distances. Pairwise distances from 0.6 to 0.8 were the most frequent and accounted for 12%–23% of all the pairwise distances (Figure 1B). Further, no genetically structured sub-populations were observed among the 250 varieties used in this study. However, several families were detected (Supplementary Figure S1).

### Distribution of Phenotypes, Genomic Heritability, and Genetic Correlation

The distributions of phenotypes (adjusted phenotypic means) based on the 250 accessions varied among the five traits studied. The distribution of Zn was almost symmetrical. The skewness was high and negative for Cu, and moderate and negative for Fe, Cd, and Mn (Supplementary Figure S2). The genomic heritability for all studied traits ranged from low (Zn: 0.14 and Cu: 0.21) to medium (Mn: 0.35) and high (Fe: 0.5 and Cd: 0.62) (Table 2). The genetic correlation estimated with the MTM model was highest between Fe and Cd (0.95) and Cu and Zn (0.95) and lowest between Mn and Cd (0.39) and Mn and Fe (0.44). Zn had the highest genetic correlations with all the other studied traits, ranging from 0.67 to 0.95 (Table 2).

**TABLE 2:** Genomic heritabilities (diagonal and bold) and genetic correlations (upper triangle) of the trace elements traits studied.

Traits	Cd	Fe	Mn	Cu	Zn
Cd	**0.62**	0.95	0.39	0.71	0.67
Fe		**0.5**	0.07	0.79	0.76
Mn			**0.35**	0.59	0.7
Cu				**0.21**	0.95
Zn					**0.14**

Diagonal and bold are genomic heritabilities as indicated.

### Prediction Accuracy of Single-Trait Model Versus Multi-Trait Model Using Whole-Genome Markers

**FIGURE 2:**
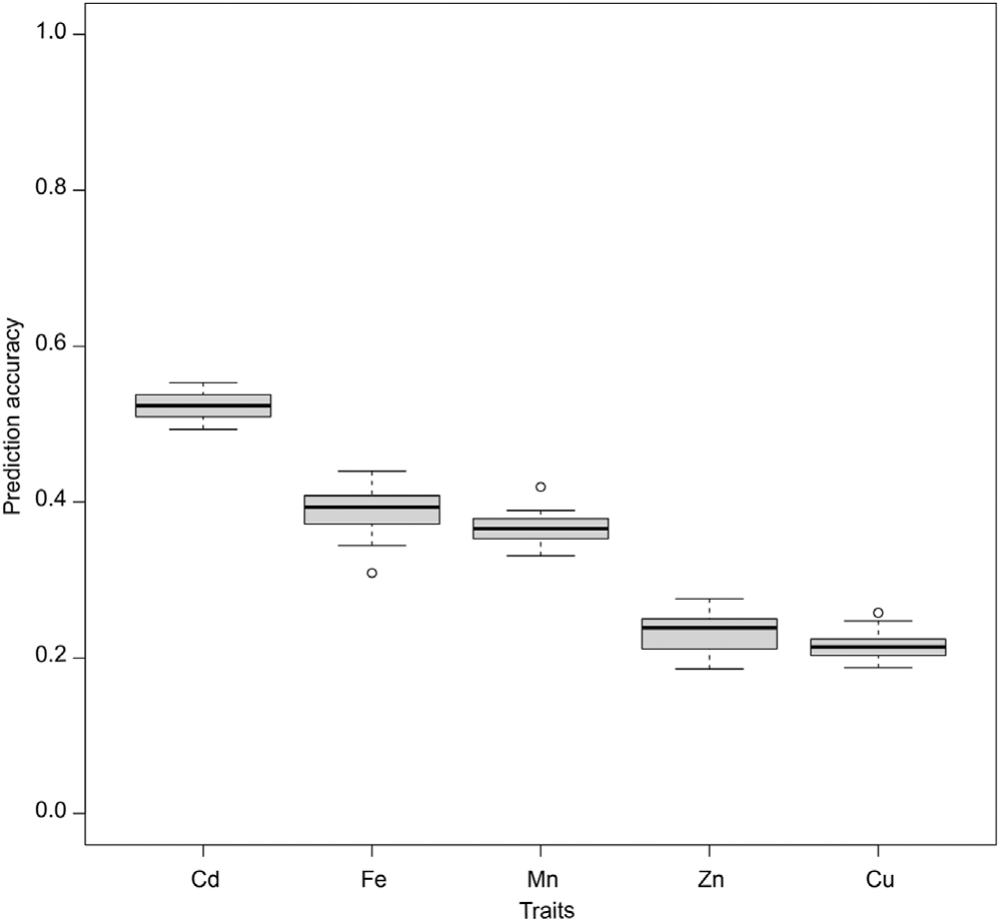
Genomic prediction accuracies of the studied traits were assessed using a single trait GBLUP (ST-GBLUP) model.

The average prediction accuracy with the traditional ST-GBLUP model under the CV1 scheme was the highest for Cd (0.52), followed by Fe (0.39), Mn (0.36), Zn (0.23), and Cu (0.21) (Figure 2). Also, under the CV1 scheme, prediction accuracies of MT models were not statistically significantly (*p <* 0.05) superior to those of ST-GBLUP irrespective of the models and traits studied (Supplementary Tables S2–S6).

**FIGURE 3:**
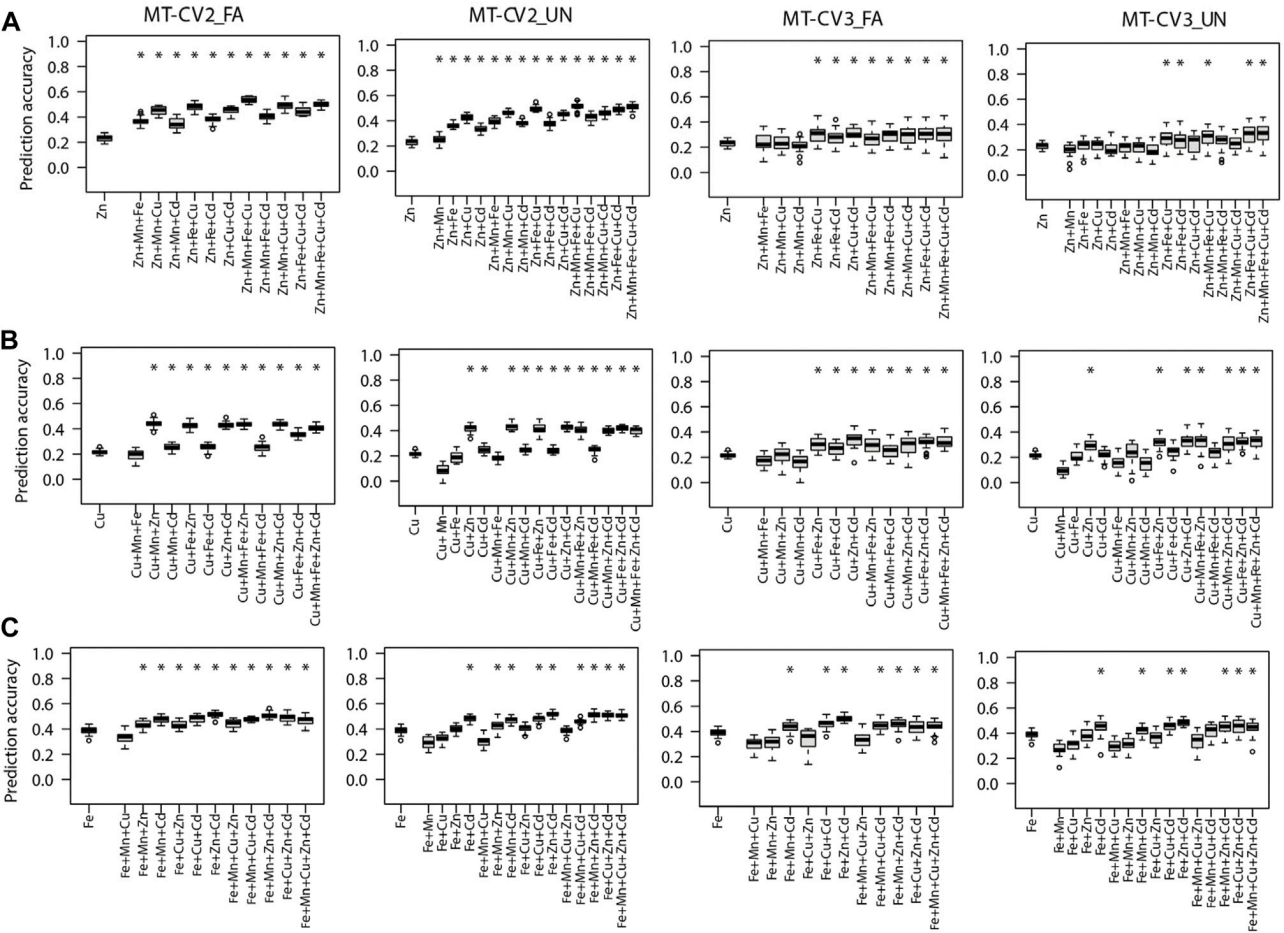
Genomic prediction accuracies of the genotype-based single trait (ST) model (ST-GBLUP) and multi-trait (MT) models (MT-FA and MT-UN) under different cross-validation (CV) schemes (ST-CV1, MT-CV2, and MT-CV3). The target traits are **(A)** Zn, **(B)** Cu, and **(C)** Fe. The first box-whisker in each portrayal indicates the accuracies of the ST-GBLUP model in the ST-CV1 scheme. Other box-whiskers refer to the accuracies achieved by MT models with different trait combinations. Asterisks above box-whiskers indicate that the prediction accuracies of the MT model for the specific trait combination were statistically significantly (*p* < 0.05, *t*-test) higher than those of the ST-GBLUP model.

**FIGURE 4:**
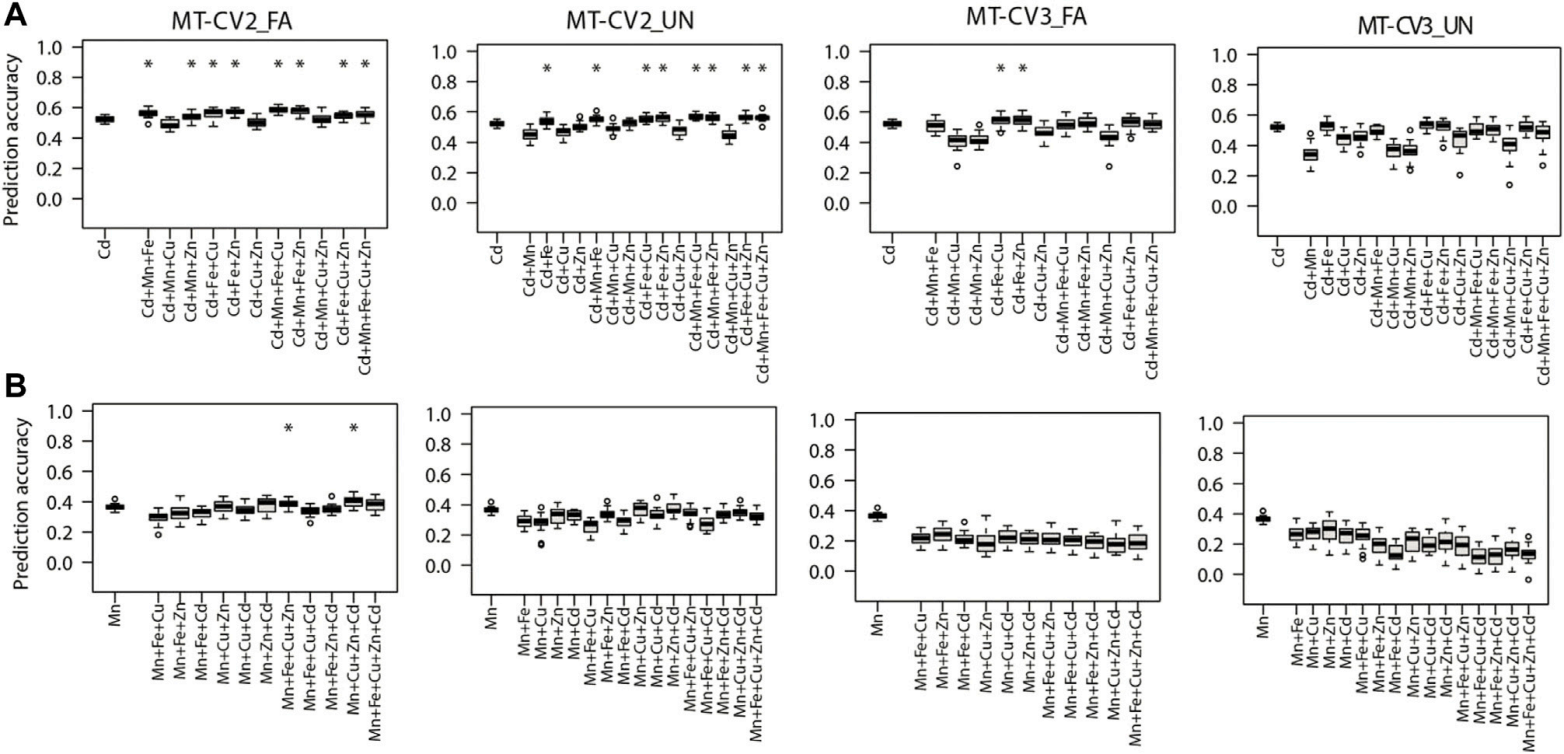
Genomic prediction accuracies of the genotype-based single trait (ST) model (ST-GBLUP) and multi-trait (MT) models (MT-FA and MT-UN) under different cross-validation (CV) schemes (ST-CV1, MT-CV2, and MT-CV3). The target traits are **(A)** Cd and **(B)** Mn. The first box-whisker in each portrayal indicates the accuracies of the ST-GBLUP model in the ST-CV1 scheme. Other box-whiskers refer to the accuracies achieved by MT models with different trait combinations. Asterisks above box-whiskers indicate that the prediction accuracies of the MT model for the specific trait combination were statistically significantly (*p* < 0.05, *t*-test) higher than those of the ST-GBLUP model.

As compared, when phenotypes of the auxiliary traits were made available in both training and test sets (MT-CV2) or merely in the test set (MT-CV3), the MT models, namely FA or UN, significantly (*p <* 0.05) outperformed the ST-GBLUP model (Figures 3, 4). For most of the studied traits, the highest performance of MT models was observed under the MT-CV2 scheme **(** Figures 3, 4 **)**. However, MT-GBLUP and BMORS MT models were not significantly (*p <* 0.05) superior to the ST-GBLUP model for all the CV schemes studied (Supplementary Tables S7–S11).

We further compared scenarios where the prediction of the target traits was assisted with a single auxiliary trait or a combination of multiple auxiliary traits in MT models. Supporting the prediction of Zn with one of its correlated traits (Cu, Fe, Mn, or Cd) was sufficient to significantly (*p* < 0.05) increase the prediction accuracy MT-UN model relative to ST-GBLUP model in MT-CV2 (Figure 3A). Cu was the best single auxiliary trait for predicting Zn. Incorporating observations from Cu in MT-UN model (under MT-CV2) significantly (*p <* 0.05) increased the prediction accuracy of Zn by 82.6% (0.23–0.42) relative to the ST-GBLUP model. However, the highest increase in prediction accuracy (126% or 0.23–0.52) of MT models was observed when observations from Mn, Fe, and Cu were combined as supporting traits for Zn under the MT-CV2 scheme (Figure 3A). Under MT-CV3, the MT-UN model outperformed the ST-GBLUP model only after multiple auxiliary traits were used to support the prediction of Zn (Figure 3A).

Similarly, compared to ST-GBLUP, the prediction accuracy of Cu by the MT-UN model significantly (*p <* 0.05) increased by 95.2% (0.21–0.41) and 38% (0.21–0.29) in MT-CV2 and MT-CV3, respectively, when Zn was used as a single supporting trait (Figure 3B). Yet, after including other traits in MT-CV2 (Mn and Zn) and MT-CV3 (Zn and Cd), the prediction accuracy improved by 109.5% (0.21–0.44) and 57.1% (0.21–0.33) relative to ST-GBLUP, respectively.

Similarly, when Fe was the target trait, MT-UN model accounting information from Cd significantly (*p <* 0.05) outperformed ST-GBLUP by 23% (0.39–0.48) and 12.8% (0.39–0.44) under MT-CV2 and MT-CV3 schemes, respectively (Figure 3C). Nevertheless, considering phenotypes from more auxiliary traits in MT-CV2 (Zn and Cd) and MT-CV3 (Zn and Cd) provided 33.3% (0.39–0.52) and 23% (0.39–0.48) gains in the prediction accuracy of Fe with the MT-UN model, respectively (Figure 3C).

Furthermore, the prediction accuracy of Cd (the most heritable trait) with the MT-UN model was significantly (*p <* 0.05) improved by 3.8% (0.52–0.54) when Fe, its strongly correlated trait, was used as a single auxiliary trait under the MT-CV2 scheme (Figure 4A). As observed for the other traits, 7.6% (0.52–0.56) and 11.5% (0.52–0.58) gains in prediction accuracy were attained after using combined information from multiple auxiliary traits (Mn, Fe, and Cu) in MT-UN and MT-FA models under MT-CV2, respectively (Figure 4A). Similarly, under MT-CV3, MT models did not significantly outperform ST-GBLUP models in scenarios where a single auxiliary trait was used. However, when information from Fe and Zn or Fe and Cu was considered, an improvement of 5.7% (0.52–0.55) in the prediction accuracy of the MT-FA model over the ST-GBLUP model was observed (Figure 4A).

Finally, when Mn was the target trait, MT-UN with a single auxiliary trait failed to improve its prediction accuracy under both MT-CV2 and MT-CV3 schemes (Figure 4B). However, considering information from additional traits (Cu, Zn, and Cd), using the MT-FA model significantly (*p <* 0.05) improved the prediction accuracy of Mn up to 11.1% (0.36–0.40) over ST-GBLUP under MT-CV2 (Figure 4B). On the other hand, under the MT-CV3 scheme, MT-UN or MT-FA did not significantly outperform ST-BLUP even after using multiple auxiliary traits.

### Prediction Accuracy of Haplotype-Based Model Versus Genotype-Based Models

**FIGURE 5:**
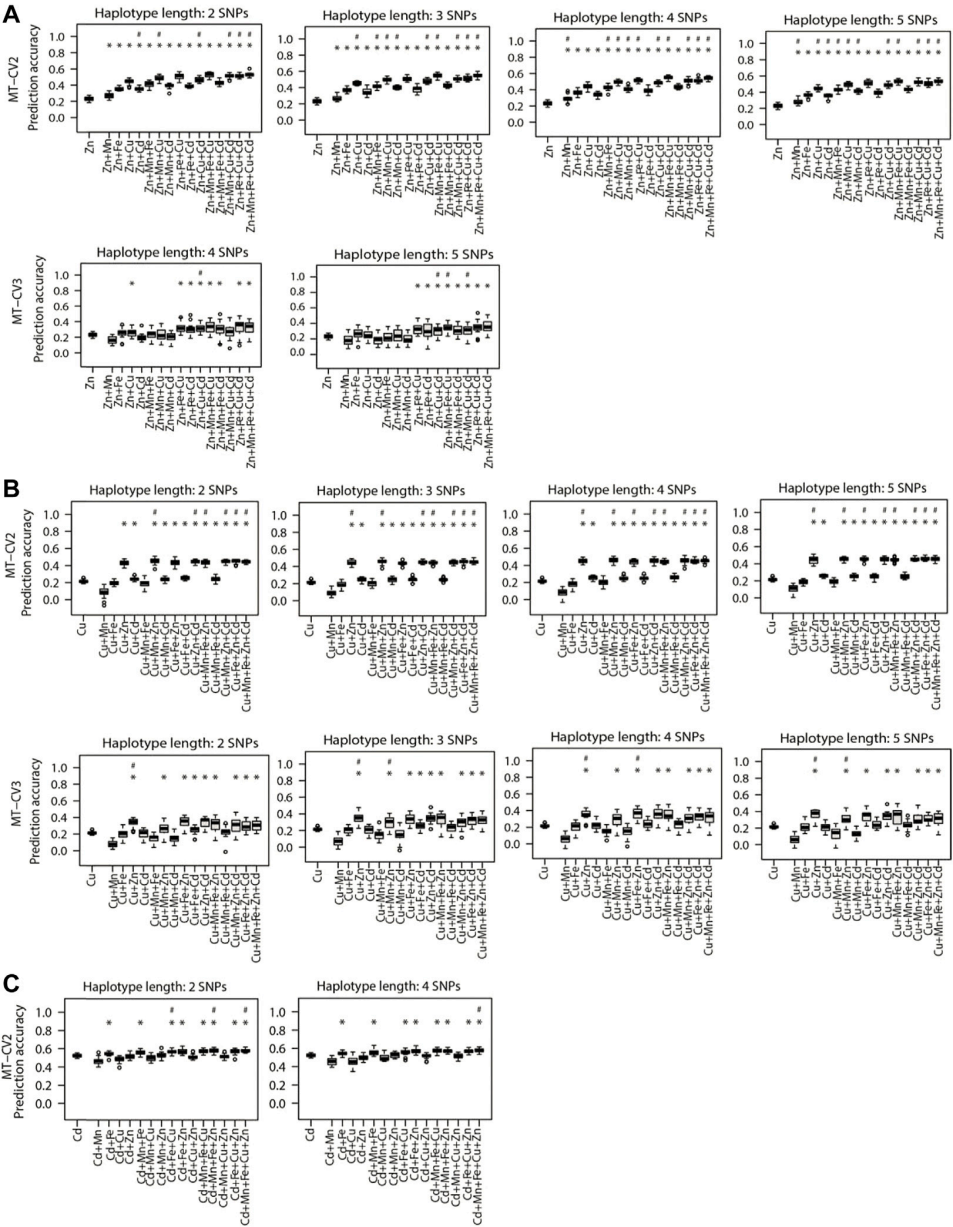
Genomic prediction accuracies of the genotype-based single trait (ST) model (ST-GBLUP) and haplotype-based multi-trait (MT) model (MT-UN) under different cross-validation (CV) schemes (ST-CV1, MT-CV2, and MT-CV3). The target traits are **(A)** Zn, **(B)** Cu, and **(C)** Cd. The number of SNPs contained in haplotype blocks ranged from three to five. The first box-whisker in each portrayal indicates the accuracies of the ST-GBLUP model in the ST-CV1 scheme. Other box-whiskers refer to the accuracies achieved by MT models with different trait combinations. Asterisks above box-whiskers indicate that the prediction accuracies of the haplotype-based MT-UN model for the specific trait combination were statistically significantly (*p* < 0.05, *t*-test) higher than those of the ST-GBLUP approach. Pounds above box-whiskers indicate that the prediction accuracies of the haplotype-based MT-UN model were statistically significantly (*p* < 0.05, *t*-test) higher than those of its corresponding genotype-based counterparts. Only the scenarios where the haplotype-based MT-UN model were statistically significantly outperformed (*p* < 0.05, *t*-test) the genotype-based MT-UN model are presented.

We further investigated the benefits of accommodating local epistatic effects on the prediction accuracy of MT models by using haplotypes instead of genotypes in the UN model. Comparing to the genotype-based UN model, the observed largest and significant (*p <* 0.05) increment of prediction accuracies using haplotype-based models was 3.8% for Zn (0.52–0.54), 4.6% for Cu (0.43–0.45), and 3.5% (0.56–0.58) for Cd under MT-CV2. For Zn, the above improvement in prediction accuracy was achieved with a haplotype length of 3 SNPs, and when Mn, Fe, Cu, and Cd were collectively used as auxiliary traits (Figure 5A). For Cu, the observed gains were realized with a haplotype length of 4 SNPs and when auxiliary traits Mn and Zn were used together (Figure 5B). For Cd, the gains were from the MT-UN model with a haplotype length of 2 SNPs, and when Mn, Fe, Cu, and Zn were combined as the auxiliary traits (Figure 5C). Under MT-CV3, the haplotype-based UN model was significantly (*p* < 0.05) superior to the genotype-based UN model by 12.5% (0.32–0.36) for Zn (Figure 5A) and 6% (0.33–0.35) for Cu (Figure 5B).

Compared to the ST-GBLUP model, the haplotype-based UN models were significantly (*p* < 0.05) superior with an increment of prediction accuracy of 134.7% (0.23–0.54), 114.2% (0.21–0.45), 23% (0.39–0.48), and 11.5% (0.52–0.58) for Zn (Figure 5A), Cu (Figure 5B), Fe (Supplementary Figure S3), and Cd (Figure 5C), respectively.

### Prediction Accuracy of a Haplotype-Based Model Capitalizing on Trait-Associated SNPs

**FIGURE 6:**
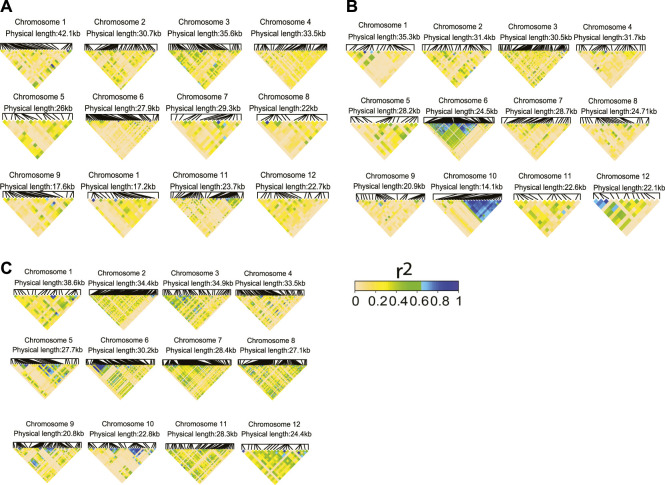
Linkage disequilibrium (LD, *r* ^2^) heatmaps for the trait-associated SNPs (TA-SNPs, *p* < 0.01) identified from a genome-wide association study (GWAS) using the total population for **(A)** Zn, **(B)** Cu, and **(C)** Cd. The physical distance indicates the distance between the first and last TA-SNPs found on each chromosome.

**FIGURE 7:**
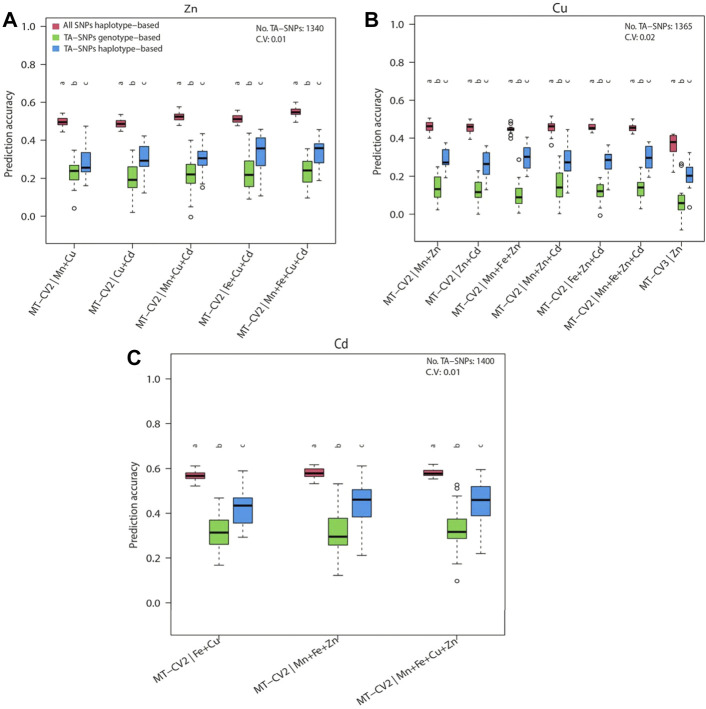
Genomic prediction accuracies of the haplotype-based multi-trait model (MT-UN) with uninformative genomic markers (all SNPs haplotype-based) and haplotype- or genotype-based multi-trait model (MT-UN) with trait-associated SNPs (TA-SNPs) under MT-CV2 and MT-CV3. The target traits were **(A)** Zn, **(B)** Cu, and **(C)** Cd. The size of the haplotype blocks containing the TA-SNPs is maximally 300 kb. Different letters above box whiskers indicate statistically significant (*p* < 0.05, *t*-test) differences among compared groups. The average number and the coefficient of variation (CV) of the used TA-SNPs for the observed predictions are shown for each trait.

With this study, we sought to investigate whether the prediction accuracy of Zn, Cu, and Cd with the haplotype-based model can be improved by using the selective marker panel made by TA-SNPs derived from GWAS (Supplementary Table S12). We purposely selected Zn and Cu because for both traits the haplotype-based MT model performed superiorly for several scenarios irrespective of the lengths of haplotypes (2–5 SNPs) (Figure 5). We also investigated Cd in addition to Zn and Cu since successive significant (*p* < 0.2) TA-SNPs were observed in GWAS based on the total population (Supplementary Figures 4A–C). The non-negligible LD (*r* ^2^≥0.1) observed between the TA-SNPs, especially in Cu and Cd, underpinned the necessity of modelling local epistatic effects among TA-SNPs (Figures 6A–C). The haplotype-based UN model accounting for TA-SNPs significantly (*p* < 0.05) outperformed their genotype-based counterparts; however, it was significantly (*p* < 0.05) inferior to the model using all genome-wide markers for all the traits and scenarios evaluated (Figures 7A–C).

## Discussion

Quantifying trace element content in food crops is labor- and time-intensive. As a result, trace element traits have been the subject of few genomic prediction studies (Owens et al., 2014; Leplat et al., 2016; Frouin et al., 2019; Guo et al., 2020; Ahmadi et al., 2021) compared to agronomic or physiological traits. This study demonstrates how MT models with appropriate CV strategies can be useful in saving phenotyping resources for trace element traits in diverse rice collections without compromising the prediction accuracy. It also provides the first proof of concept in diverse rice for incorporating local epistatic effects and trait-associated SNPs into MT genomic prediction models.

### Multi-Trait Models Improved the Prediction Accuracy of Trace Elements in Rice Grain

In this study, MT models did not significantly outperform ST-GBLUP under the CV1 scheme for all the scenarios evaluated (Supplementary Tables S2–S6). Earlier studies also reported insignificant differences in the prediction accuracies of MT-CV1 and ST-CV1 (Calus and Veerkamp, 2011; dos Santos et al., 2016; Bhatta et al., 2020), implying that MT models are not always robust over ST models, especially when information on auxiliary traits is only available in the training set and the unobserved accessions are predicted only based on genotypic data. In contrast, when phenotypes of the auxiliary traits were present in the training and test set (MT-CV2) or merely in the test set (MT-CV3), the prediction accuracy of MT models (MT-UN and MT-FA) for the unobserved target traits (Zn, Cu, Fe, and Cd) was significantly improved relative to ST-GBLUP (Figures 3, 4). Previous studies attributed the predictive performance of MT models to both higher heritability of the auxiliary trait and strong genetic correlation between the target and auxiliary traits (Sun et al., 2017; Fernandes et al., 2018).

### Accounting for the Information From Multiple Auxiliary Traits Boosted the Predictive Ability of Multi-Trait Models

Using a single auxiliary trait in the MT-UN model significantly (*p* < 0.05) improved the prediction accuracy of target traits Zn, Cu, Fe, and Cd relative to the ST-GBLUP model (Figures 3, 4). When a strong genetic correlation exists between target and auxiliary trait, the prediction accuracy of MT models could still be improved under MT-CV2 or MT-CV3 regardless of trait heritability. For instance, supporting the prediction of Cu with Zn, its strongly correlated trait (cor _Zn, Cu_ = 0.95) but with lower heritability (h^2^_Zn_ = 0.14; h^2^_Cu_ = 0.21) significantly improved the prediction accuracy of Cu with the MT-UN model (Figure 3B). Also, supporting Cd with Fe, its strongly correlated trait (cor _Cd, Fe_ = 0.95) but with lower heritability (h^2^_Cd_ = 0.62; h^2^_Fe_ = 0.50), improved the prediction accuracy of Cd with the MT-UN model (Figure 4A). Arojju et al. (2020) also indicated that the genetic correlation was the main cause of the observed gain in prediction accuracy of MT models. The same study further showed that when a trait in strong genetic correlation with the target trait is used in the MT model, the predictive performance of the MT model was still superior to the ST model even after reducing the training population size by 50%.

Collectively accounting for phenotypes of multiple auxiliary traits further improved the predictive ability of the MT models compared to the MT models with a single auxiliary trait. For example, the highest increase in the prediction of Zn was 82.6% when a single auxiliary trait was used in MT-UN models. Yet, using multiple traits collectively in the same model improved the prediction of Zn by 126% compared to the ST-GBLUP model (Figure 3A). Also, MT models with one auxiliary trait showed no benefit over the ST-GBLUP model when predicting Mn, with relative medium heritability and no strong genetic correlation with any other studied trait. However, when auxiliary traits were collectively used in the MT-FA model, significant improvements in the prediction accuracy over the ST-GBLUP model were observed (Figure 4B). Multiple auxiliary traits would optimize MT models, though the assisting trait *per se* is neither strongly genetically correlated with the target trait nor highly heritable. Therefore, when no single auxiliary trait meets the criterion of heritability or genetic correlation, combining multiple auxiliary traits in the MT model could be an effective approach to enhance the predictive ability of MT models. These findings are concurrent with previous findings by Wang et al. (2017), indicating that the prediction accuracy of MT models was highest when eight different traits were used as auxiliary traits to predict grain yield in rice.

### Modeling Local Epistatic Effects is Beneficial in Multi-Trait Models Irrespective of Using Total or Selective Marker Panel

Previous studies demonstrated that accounting for local epistatic effects besides the additive effect in genomic prediction could improve the prediction accuracy of ST models (Akdemir and Jannink, 2015; Akdemir et al., 2017; Jiang et al., 2018; He et al., 2019). Here, we are the first to attempt to model the local epistatic effects in the context of MT genomic prediction. Accounting for the local epistatic effects in haplotype-based MT models significantly improved the prediction accuracy of Zn, Cu, and Cd relative to genotype-based MT models, only capturing additive effects (Figures 5A–C). Relative to ST-GLUP, the highest increase in prediction accuracy, 134.7% for Zn, was observed after incorporating the local epistatic effects into the MT-UN model (Figure 5A). These findings imply that the potential of MT models can be maximized by accounting for local epistatic effects besides additive effects in the model.

Using a selective marker panel based on approaches exploiting the trait biological and genetic background knowledge such as GWAS has been proven effective to improve the predictive ability of GS models (Owens et al., 2014; Wang et al., 2019). Our study did not show any improvements of prediction accuracy by using the TA-SNPs instead of all genome-wide SNPs for Cu, Zn, and Cd (Figures 7A–C). These findings could be attributed to the complex genetic architectures of the trace elements we studied (Supplementary Figures S4A–C). Our approach was slightly similar to previous methods using GWAS-derived TA-SNPs to construct the trait-associated matrix (Zhang et al., 2014; Ahmadi et al., 2021), except that we did not assign weights to haplotype- or genotype-based genomic relationship matrices. Though numerous studies reported improved gains from using the above strategy (Bhandari et al., 2019; Ahmadi et al., 2021), Veerkamp et al. (2016) showed that the proportion of total variance explained by the TA-SNPs combined in a GRM was considerably smaller than that explained by all variants in Holstein-Friesian cattle population. A potentially more promising way to use TA-SNPs would be to fit them as fixed effects in the GP model along with all other SNPs as random effects (Spindel et al., 2016). However, the latter approach is best suited for features with a few large-effect QTLs in a polygenic context (Poland and Rutkoski, 2016; Bian and Holland, 2017; Rice and Lipka, 2019). Therefore, the genetic architecture of the target traits must be studied before applying this strategy to a breeding program. The marked advantage of the haplotype-based UN model over their genotype-based counterparts using the TA-SNPs (Figures 7A–C) substantiates the existence of local epistasis in trace element traits (Sharma V. et al., 2021) and the merit of modelling local epistatic effects in MT-GP program.

### Factors Affecting the Observed Prediction Accuracies: Trait Heritability, Genetic Correlation, and Population Relatedness

Various factors affect the predictive ability of GP models used in GS (Crossa et al., 2017; Xu et al., 2021). In this study, the genomic heritabilities spanned a wide range from 0.14 to 0.62 (Table 2), which enabled the evaluation of the performance of MT models under contrasting levels of genomic heritability. The genomic heritability of Zn was the lowest (h^2^ = 0.14, Table 2), which contradicts several previous studies that reported moderate to high heritability of Zn (Norton et al., 2010; Pinson et al., 2015; Naik et al., 2020). The poor heritability estimate of Zn in this study could be due to potential environmental effects. Unfortunately, our study does not include multi-environment trials and therefore does not provide insight into environmental factors and GxE interactions on genomic prediction of trace element traits.

Strong genetic correlations (Cor > 0.75) were observed between pairs of the studied trace element traits (Table 2). This was expected due to their overlapping genetic and physiological mechanisms (Sasaki et al., 2012; Cu et al., 2020). For example, transporter gene families like zinc-iron permease (ZIP), natural resistance-associated macrophage proteins (NRAMPs), and heavy metal transporting, ATPases (HMAs) have been associated with uptake and translocation of several trace elements in plants (Fernández-Paz et al., 2021; Vanderschueren et al., 2021; Zhang et al., 2022). As a result, borrowing information from correlated traits overall improved the prediction accuracy of the MT models.

The success of GS is also highly dependent on the LD between markers and unknown causal variants. The genetically distant training and test sets would have different LD decay patterns and consequently impede the prediction (Snelling et al., 2013; Desta and Ortiz, 2014; Thistlethwaite et al., 2020). Such a problem is typically prominent in germplasm accessions, limiting the power of GP (Crossa et al., 2017). This is also the case for the diverse rice population used in this study, as most accession pairs were distantly unrelated (Figure 1B). As a result, the predictive ability for ST-GBLUP was poor (Figure 2), particularly for traits with the lowest estimated heritabilities, namely Zn and Cu (Table 2). Adding related materials to the training population has been suggested to overcome the problem of low relatedness between training and test populations and improve the accuracy of genomic prediction (Arenas et al., 2021). Nevertheless, increasing relatedness will damage genetic gain in the long term because genetic variation will be limited or exhausted if related populations are overused (Jannink et al., 2010; Moeinizade et al., 2019). We show that MT models are powerful tools for predicting trace element traits in populations with diverse backgrounds. However, further studies with larger datasets are needed to elucidate the utility of different populations and marker optimization strategies in the context of MT genomic prediction.

### The Prospect of Multi-Trait Models for the Evaluation of Genetic Resources in Gene Banks

**FIGURE 8:**
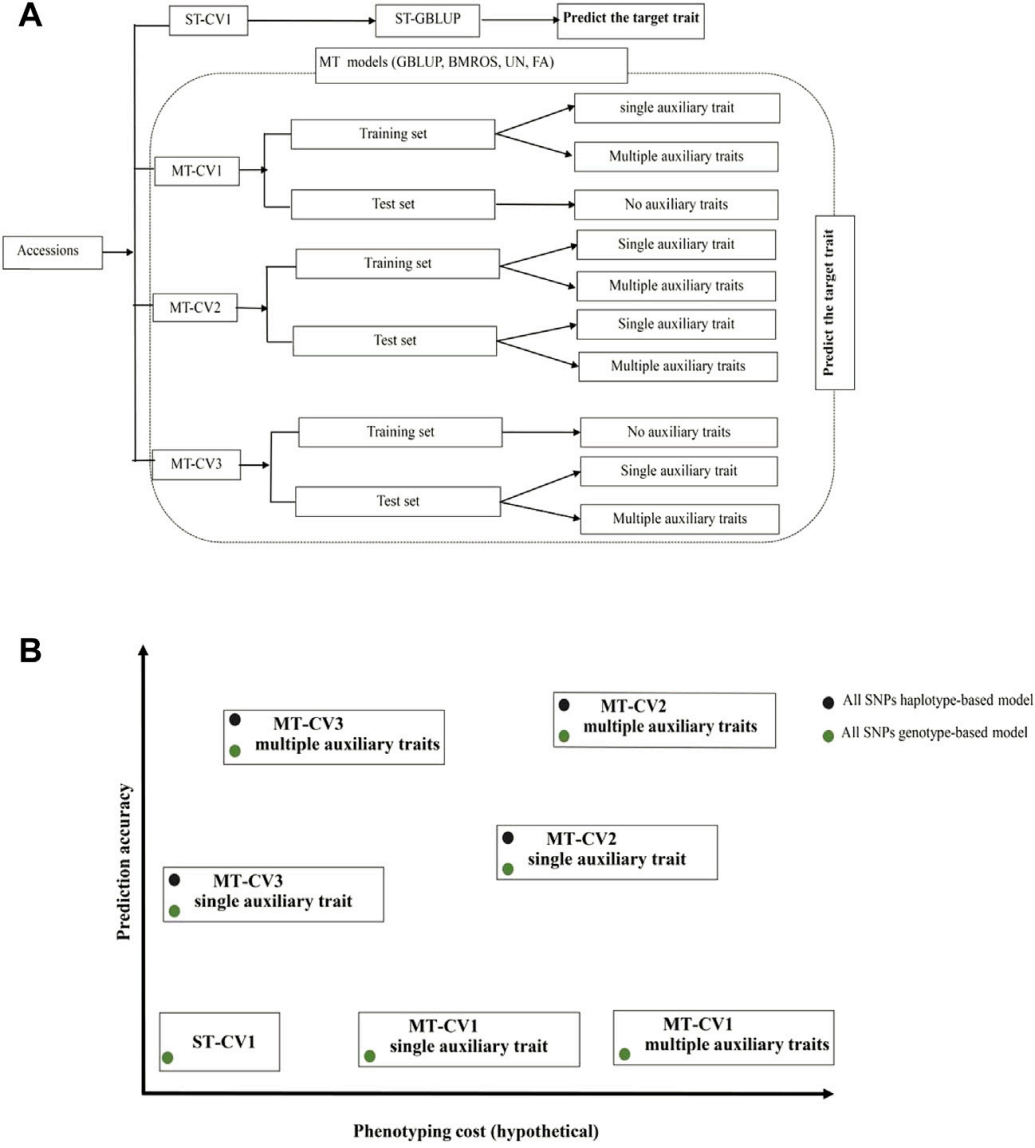
General recommendations for using cross-validation (CV) schemes and multi-trait (MT) models. **(A)** An illustration of the different CV partitions and trait combination scenarios evaluated. **(B)** Expected prediction accuracy and phenotyping cost for different CV schemes and MT models. Green dots represent models which account only for additive effects. Black dots represent models considering both additive and local epistatic effects. The GBLUP model under ST-CV1 is economically advantageous because the main effort is just devoted to phenotyping one target trait in the training set. However, in terms of prediction accuracy, it is less robust than the UN and FA MT models under MT-CV2 or MT-CV3. Compared to ST-GBLUP, using MT models under MT-CV1 has no advantage in phenotypic resource-saving or prediction accuracy. In contrast, MT models implemented under MT-CV2 and MT-CV3 can improve prediction accuracy. However, high phenotyping efforts can be expected with MT-CV2, mainly when multiple auxiliary traits need to be phenotyped. MT-CV3 saves resources by only phenotyping the test set population (20% of the total population in our case). Accounting for local epistatic effects may further improve the predictive ability of MT models under MT-CV2 or MT-CV3.

Expediting genomic selection in gene banks to predict the genetic merit of the unobserved accessions would enable accurate identification of promising donor accessions without a comprehensive phenotypic test of all the accessions in the field (Pace et al., 2015; Yu et al., 2016; Crossa et al., 2017; Tanaka et al., 2021; He et al., 2022). In fact, as the traits of breeders’ interest are extensive, the genetic resources archived in gene banks would be evaluated for several traits. MT genomic prediction is an effective method to realize this comprehensive evaluation. Our study tested several MT models under three different CV schemes, implying different phenotyping layouts and costs. We found that high prediction accuracy of MT models can be achieved under prediction schemes MT-CV2 and MT-CV3. The MT-CV2 scheme requires more budget for phenotyping auxiliary traits in both training and test sets. Therefore, breeders may kindly MT-CV2 if phenotypes for the auxiliary traits can be inexpensively obtained. Otherwise, MT-CV3 is more cost-effective as phenotypes for the auxiliary traits are only required for the test set (i.e., 20% of the entire population). Since using multiple auxiliary traits collectively in the MT model can improve prediction even if the individual auxiliary traits do not fully meet the heritability and genetic correlation conditions, an ideal situation would be to phenotype less expensive and more manageable traits (e.g., root system architecture, 100-grain weight, data to heading, etc.) to support the prediction of expensive target traits with the MT models. Besides, accounting for local epistatic effects in MT models would help to improve the predictive ability. The different scenarios studied here and their respective potentials in terms of prediction accuracy and phenotyping cost are illustrated in Figures 8A,B.

To breed safe and nutritious crop varieties, further studies using the genomic selection index (Habyarimana et al., 2020), for example, are desired to provide a comprehensive understanding of the strategies to optimize essential nutrients and toxic metals such as Cd in food crops.

## Data Availability

The original contributions presented in the study are included in the article/Supplementary Material; further inquiries can be directed to the corresponding authors.
