# Supplementary material for: Multi-Trait Genomic Prediction Models Enhance the Predictive Ability of Grain Trace Elements in Rice
Source: Front Genet. 2022 Jun 22;13:883853. doi: 10.3389/fgene.2022.883853 (PMC9257107; doi:10.3389/fgene.2022.883853)
Supplement: Supplementary file 3 [file DataSheet2.docx]

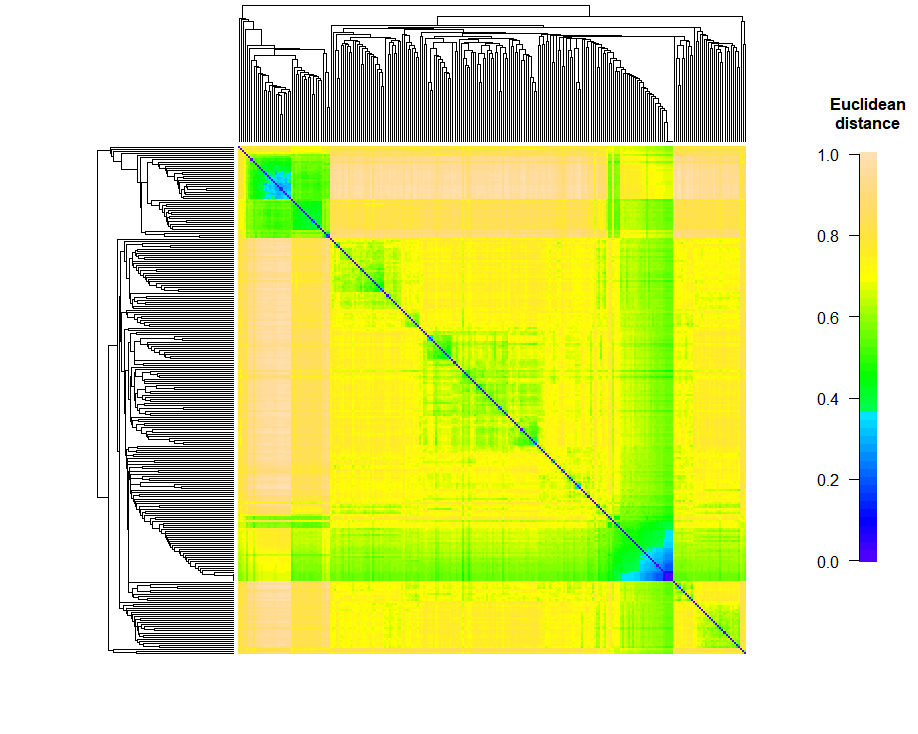


**Supplementary Figure 1** Pairwise genetic dissimilarities among 250 rice varieties described by Euclidean distance. The average cluster method was used to order the accessions.


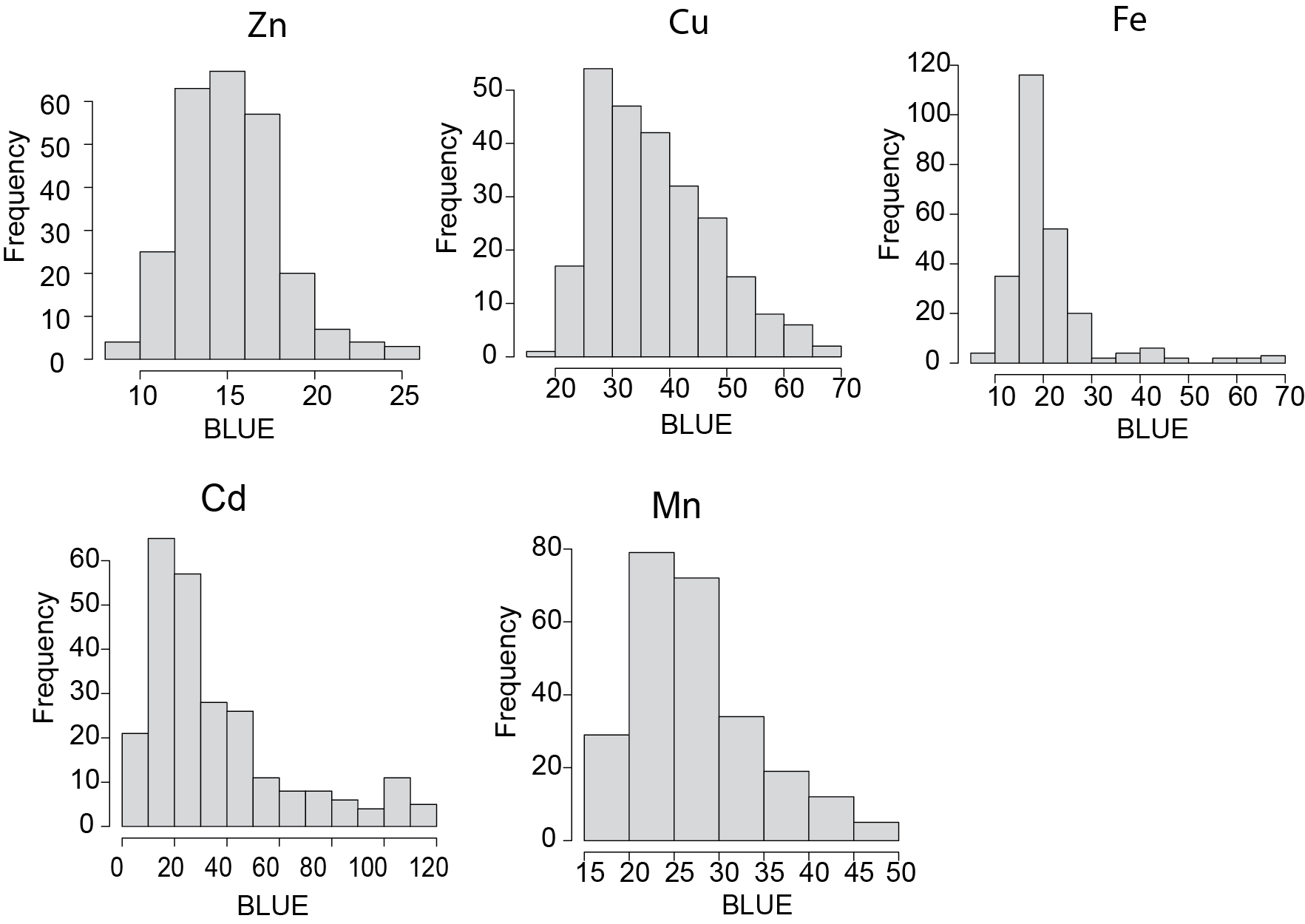


**Supplementary Figure 2** Distribution of phenotypic data for Zn, Cu, Fe, Cd, and Mn.

**
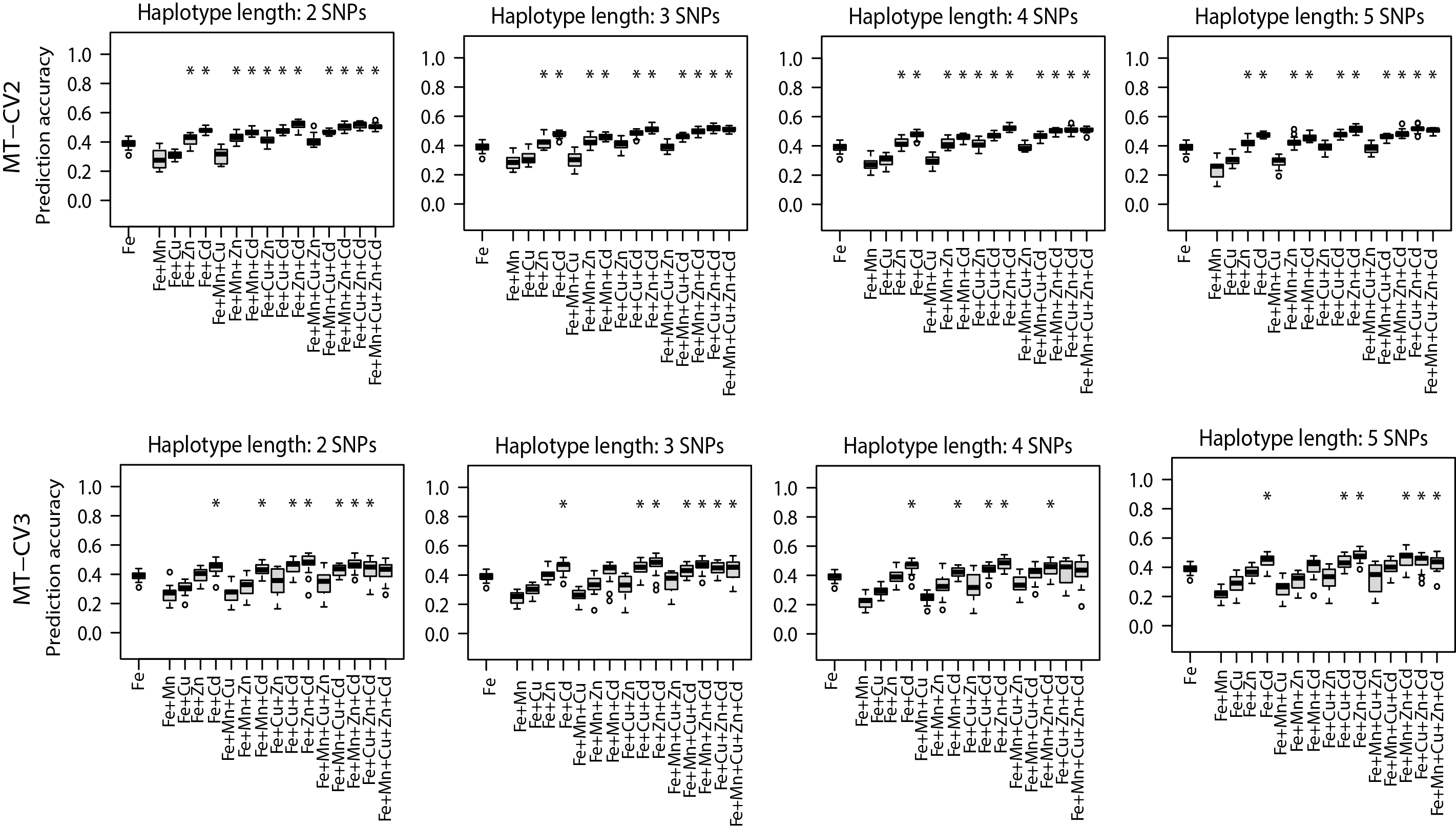
**

**Supplementary Figure 3** Genomic prediction accuracies of Fe using genotype-based single trait (ST) model (ST-GBLUP) and haplotype-based multi-trait (MT) model (MT-UN) under different cross-validation (CV) schemes (ST-CV1, MT-CV2 and MT-CV3). The number of SNPs included in the haplotype model ranged from two to five. The first box-whisker in each portrayal indicates the accuracies of ST-GBLUP model in ST-CV1 scheme. Other box-whiskers refer to the accuracies achieved by MT-UN model with different trait combinations under MT-CV2 and MT-CV3 schemes. Asterisks above box-whiskers indicate that the prediction accuracies of the haplotype-based MT-UN model for the specific trait combination was statistically significantly (*p* < 0.05, t-test) higher than those of ST-GBLUP model. Only the scenarios where MT-UN model showed any statistically significant improvements in prediction accuracy relative to ST-GBLUP were presented.

**
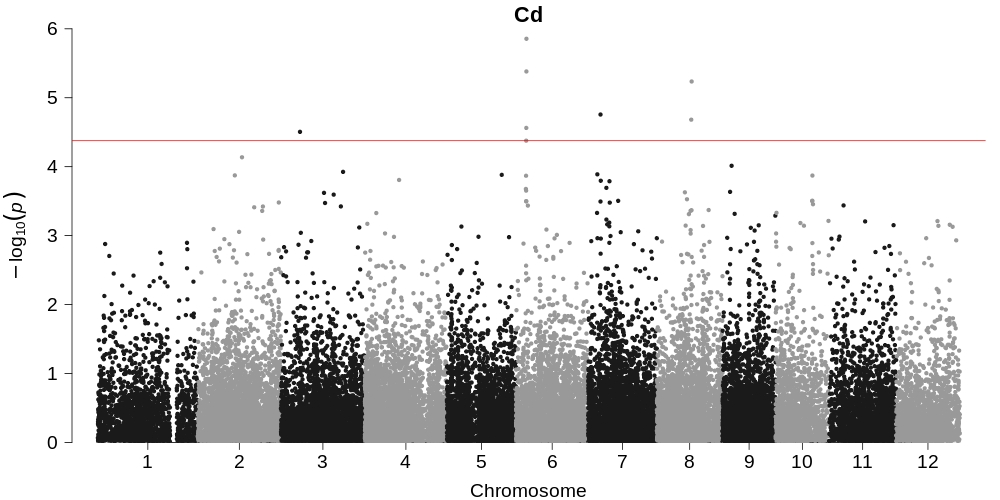
**

A

B

**
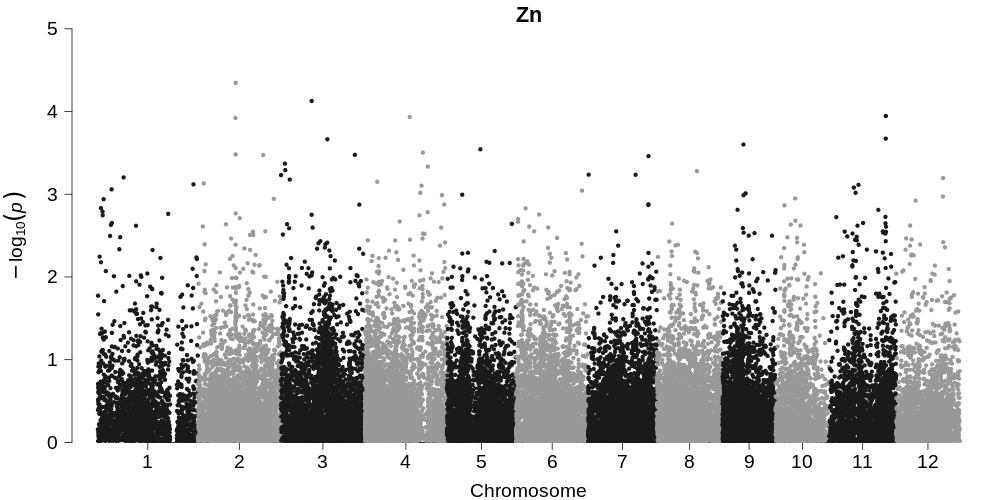
**

C

**
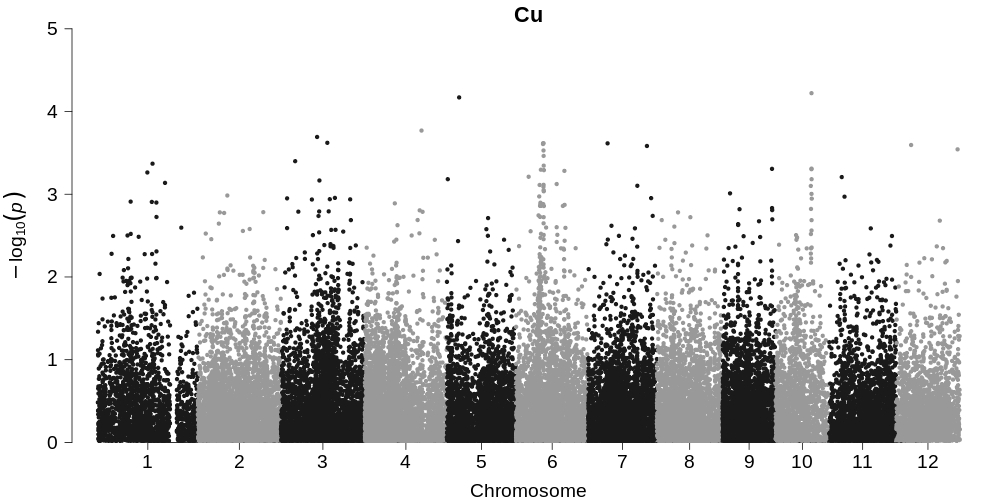
**

**Supplementary Figure 4** Manhattan plots for the genome-wide association study (GWAS) of (A) Cd, (B) Zn, and (C) Cu. The red horizontal line indicates a genome-wide significance threshold of 0.2 when *p* values are adjusted by FDR
