# Supplementary material for: Multi-Trait Genomic Prediction Models Enhance the Predictive Ability of Grain Trace Elements in Rice
Source: Front Genet. 2022 Jun 22;13:883853. doi: 10.3389/fgene.2022.883853 (PMC9257107; doi:10.3389/fgene.2022.883853)
Supplement: Supplementary file 4 [file DataSheet1.docx]

**Supplementary Table 2** Average prediction accuracies of Zn using single trait (ST) and multi-trait (MT) genomic prediction models under cross-validation scheme one (CV1). The highest prediction accuracy of MT models is underlined.

| CV scheme | Trait combinations | ST-GBLUP | MT-GBLUP | BMORS | FA^§^ | UN |
| --- | --- | --- | --- | --- | --- | --- |
| ST-CV1 | Zn | 0.23 |  |  |  |  |
| MT_CV1 | Zn+Mn |  | 0.20 | 0.13 |  | 0.16 |
|  | Zn+Fe |  | 0.20 | 0.15 |  | 0.16 |
|  | Zn+Cu |  | 0.19 | 0.16 |  | 0.16 |
|  | Zn+Cd |  | 0.19 | 0.15 |  | 0.19 |
|  | Zn+Mn+Fe |  | 0.19 | 0.17 | 0.16 | 0.15 |
|  | Zn+Mn+Cu |  | 0.20 | 0.16 | 0.14 | 0.15 |
|  | Zn+Mn+Cd |  | 0.18 | 0.13 | 0.16 | 0.16 |
|  | Zn+Fe+Cu |  | 0.20 | 0.16 | 0.16 | 0.14 |
|  | Zn+Fe+Cd |  | 0.20 | 0.14 | 0.18 | 0.17 |
|  | Zn+Cu+Cd |  | 0.18 | 0.15 | 0.18 | 0.15 |
|  | Zn+Mn+Fe+Cu |  | 0.19 | 0.14 | 0.14 | 0.15 |
|  | Zn+Mn+Fe+Cd |  | 0.20 | 0.14 | 0.18 | 0.14 |
|  | Zn+Mn+Cu+Cd |  | 0.20 | 0.16 | 0.15 | 0.16 |
|  | Zn+Fe+Cu+Cd |  | 0.21 | 0.14 | 0.16 | 0.16 |
|  | Zn+Mn+Fe+Cu+Cd |  | 0.18 | 0.16 | 0.15 | 0.16 |

^§^ FA model requires at least two auxiliary traits.

**Supplementary Table 3** Average prediction accuracies of Cu using single trait (ST) and multi-trait (MT) genomic prediction models under cross-validation scheme one (CV1). The highest prediction accuracy of MT models is underlined.

| CV scheme | Trait combinations | ST-GBLUP | MT-GBLUP | BMORS | FA^§^ | UN |
| --- | --- | --- | --- | --- | --- | --- |
| ST-CV1 | Cu | 0.21 |  |  |  |  |
| MT_CV1 | Cu+Mn |  | 0.08 | 0.15 |  | 0.08 |
|  | Cu+Fe |  | 0.09 | 0.13 |  | 0.14 |
|  | Cu+Zn |  | 0.10 | 0.13 |  | 0.16 |
|  | Cu+Cd |  | 0.10 | 0.12 |  | 0.17 |
|  | Cu+Mn+Fe |  | 0.08 | 0.13 | 0.14 | 0.10 |
|  | Cu+Mn+Zn |  | 0.10 | 0.13 | 0.15 | 0.15 |
|  | Cu+Mn+Cd |  | 0.08 | 0.14 | 0.16 | 0.13 |
|  | Cu+Fe+Zn |  | 0.07 | 0.14 | 0.17 | 0.15 |
|  | Cu+Fe+Cd |  | 0.09 | 0.15 | 0.17 | 0.15 |
|  | Cu+Zn+Cd |  | 0.08 | 0.16 | 0.15 | 0.16 |
|  | Cu+Mn+Fe+Zn |  | 0.06 | 0.13 | 0.14 | 0.14 |
|  | Cu+Mn+Fe+Cd |  | 0.08 | 0.13 | 0.16 | 0.12 |
|  | Cu+Mn+Zn+Cd |  | 0.07 | 0.15 | 0.16 | 0.15 |
|  | Cu+Fe+Zn+Cd |  | 0.07 | 0.14 | 0.17 | 0.14 |
|  | Cu+Mn+Fe+Zn+Cd |  | 0.07 | 0.14 | 0.15 | 0.12 |

^§^ FA model requires at least two auxiliary traits.

**Supplementary Table 4** Average prediction accuracies of Fe using single trait (ST) and multi-trait (MT) genomic prediction models under cross-validation scheme one (CV1). The highest prediction accuracy of MT models is underlined.

| CV scheme | Trait combinations | ST-GBLUP | MT-GBLUP | BMORS | FA^§^ | UN |
| --- | --- | --- | --- | --- | --- | --- |
| ST-CV1 | Fe | 0.39 |  |  |  |  |
| MT_CV1 | Fe+Mn |  | 0.20 | 0.27 |  | 0.31 |
|  | Fe+Cu |  | 0.20 | 0.24 |  | 0.31 |
|  | Fe+Zn |  | 0.18 | 0.27 |  | 0.30 |
|  | Fe+Cd |  | 0.19 | 0.26 |  | 0.34 |
|  | Fe+Mn+Cu |  | 0.21 | 0.27 | 0.30 | 0.30 |
|  | Fe+Mn+Zn |  | 0.19 | 0.27 | 0.29 | 0.29 |
|  | Fe+Mn+Cd |  | 0.20 | 0.29 | 0.35 | 0.34 |
|  | Fe+Cu+Zn |  | 0.21 | 0.27 | 0.28 | 0.25 |
|  | Fe+Cu+Cd |  | 0.18 | 0.28 | 0.35 | 0.35 |
|  | Fe+Zn+Cd |  | 0.20 | 0.27 | 0.33 | 0.33 |
|  | Fe+Mn+Cu+Zn |  | 0.20 | 0.27 | 0.29 | 0.23 |
|  | Fe+Mn+Cu+Cd |  | 0.18 | 0.26 | 0.34 | 0.33 |
|  | Fe+Mn+Zn+Cd |  | 0.18 | 0.27 | 0.33 | 0.33 |
|  | Fe+Cu+Zn+Cd |  | 0.19 | 0.29 | 0.30 | 0.33 |
|  | Fe+Mn+Cu+Zn+Cd |  | 0.19 | 0.27 | 0.30 | 0.32 |

^§^ FA model requires at least two auxiliary traits.

**Supplementary Table 5** Average prediction accuracies of Cd using single trait (ST) and multi-trait (MT) genomic prediction models under cross-validation (CV) scheme CV1. The highest prediction accuracy of MT models is underlined.

| CV scheme | Trait combinations | ST-GBLUP | MT-GBLUP | BMORS | FA**^§^** | UN |
| --- | --- | --- | --- | --- | --- | --- |
| ST-CV1 | Cd | 0.52 |  |  |  |  |
| MT-CV1 | Cd+Mn |  | 0.40 | 0.37 |  | 0.42 |
|  | Cd+Fe |  | 0.40 | 0.37 |  | 0.44 |
|  | Cd+Cu |  | 0.41 | 0.36 |  | 0.43 |
|  | Cd+Zn |  | 0.40 | 0.35 |  | 0.42 |
|  | Cd+Mn+Fe |  | 0.40 | 0.37 | 0.45 | 0.44 |
|  | Cd+Mn+Cu |  | 0.40 | 0.36 | 0.42 | 0.42 |
|  | Cd+Mn+Zn |  | 0.38 | 0.36 | 0.42 | 0.44 |
|  | Cd+Fe+Cu |  | 0.38 | 0.38 | 0.45 | 0.45 |
|  | Cd+Fe+Zn |  | 0.40 | 0.38 | 0.44 | 0.43 |
|  | Cd+Cu+Zn |  | 0.39 | 0.35 | 0.43 | 0.41 |
|  | Cd+Mn+Fe+Cu |  | 0.40 | 0.37 | 0.44 | 0.44 |
|  | Cd+Mn+Fe+Zn |  | 0.40 | 0.37 | 0.43 | 0.43 |
|  | Cd+Mn+Cu+Zn |  | 0.39 | 0.36 | 0.42 | 0.37 |
|  | Cd+Fe+Cu+Zn |  | 0.41 | 0.38 | 0.43 | 0.42 |
|  | Cd+Mn+Fe+Cu+Zn |  | 0.40 | 0.35 | 0.43 | 0.41 |

**^§^** FA model requires at least two auxiliary traits.

**Supplementary Table 6** Average prediction accuracies of Mn using single trait (ST) and multi-trait (MT) genomic prediction models under cross-validation scheme one (CV1). The highest prediction accuracy of MT models is underlined.

| CV scheme | Trait combinations | ST-GBLUP | MT-GBLUP | BMORS | FA**^§^** | UN |
| --- | --- | --- | --- | --- | --- | --- |
| ST-CV1 | Mn | 0.36 |  |  |  |  |
| MT-CV1 | Mn+Fe |  | 0.25 | 0.19 |  | 0.27 |
|  | Mn+Cu |  | 0.25 | 0.19 |  | 0.27 |
|  | Mn+Zn |  | 0.24 | 0.20 |  | 0.26 |
|  | Mn+Cd |  | 0.23 | 0.17 |  | 0.27 |
|  | Mn+Fe+Cu |  | 0.24 | 0.20 | 0.29 | 0.27 |
|  | Mn+Fe+Zn |  | 0.24 | 0.19 | 0.28 | 0.27 |
|  | Mn+Fe+Cd |  | 0.25 | 0.19 | 0.28 | 0.27 |
|  | Mn+Cu+Zn |  | 0.25 | 0.20 | 0.27 | 0.26 |
|  | Mn+Cu+Cd |  | 0.25 | 0.19 | 0.28 | 0.28 |
|  | Mn+Zn+Cd |  | 0.26 | 0.18 | 0.28 | 0.26 |
|  | Mn+Fe+Cu+Zn |  | 0.25 | 0.20 | 0.27 | 0.25 |
|  | Mn+Fe+Cu+Cd |  | 0.23 | 0.20 | 0.28 | 0.25 |
|  | Mn+Fe+Zn+Cd |  | 0.24 | 0.18 | 0.27 | 0.26 |
|  | Mn+Cu+Zn+Cd |  | 0.23 | 0.18 | 0.27 | 0.25 |
|  | Mn+Fe+Cu+Zn+Cd |  | 0.25 | 0.19 | 0.27 | 0.21 |

**^§^** FA model requires at least two auxiliary traits.

**Supplementary Table 7** Average genomic prediction accuracies of Zn using single trait (ST) model (ST-GBLUP) and multi-trait (MT) models (MT-GBLUP and BMORS) under different cross-validation (CV) schemes. The highest prediction accuracy of MT models is underlined.

| CV scheme | Trait combinations | ST-GBLUP | MT-GBLUP | BMORS |
| --- | --- | --- | --- | --- |
| ST-CV1 | Zn | 0.23 |  |  |
| MT_CV2 | Zn+Mn |  | 0.19 | 0.15 |
|  | Zn+Fe |  | 0.19 | 0.16 |
|  | Zn+Cu |  | 0.20 | 0.2 |
|  | Zn+Cd |  | 0.19 | 0.18 |
|  | Zn+Mn+Fe |  | 0.21 | 0.19 |
|  | Zn+Mn+Cu |  | 0.19 | 0.21 |
|  | Zn+Mn+Cd |  | 0.18 | 0.18 |
|  | Zn+Fe+Cu |  | 0.20 | 0.23 |
|  | Zn+Fe+Cd |  | 0.19 | 0.18 |
|  | Zn+Cu+Cd |  | 0.21 | 0.21 |
|  | Zn+Mn+Fe+Cu |  | 0.20 | 0.24 |
|  | Zn+Mn+Fe+Cd |  | 0.20 | 0.20 |
|  | Zn+Mn+Cu+Cd |  | 0.19 | 0.24 |
|  | Zn+Fe+Cu+Cd |  | 0.18 | 0.23 |
|  | Zn+Mn+Fe+Cu+Cd |  | 0.18 | 0.24 |
| MT_CV3 | Zn+Mn |  | 0.20 | 0.13 |
|  | Zn+Fe |  | 0.18 | 0.09 |
|  | Zn+Cu |  | 0.19 | 0.10 |
|  | Zn+Cd |  | 0.20 | 0.14 |
|  | Zn+Mn+Fe |  | 0.19 | 0.08 |
|  | Zn+Mn+Cu |  | 0.19 | 0.09 |
|  | Zn+Mn+Cd |  | 0.20 | 0.13 |
|  | Zn+Fe+Cu |  | 0.18 | 0.08 |
|  | Zn+Fe+Cd |  | 0.19 | 0.13 |
|  | Zn+Cu+Cd |  | 0.19 | 0.12 |
|  | Zn+Mn+Fe+Cu |  | 0.20 | 0.05 |
|  | Zn+Mn+Fe+Cd |  | 0.19 | 0.10 |
|  | Zn+Mn+Cu+Cd |  | 0.18 | 0.09 |
|  | Zn+Fe+Cu+Cd |  | 0.20 | 0.10 |
|  | Zn+Mn+Fe+Cu+Cd |  | 0.20 | 0.07 |

**Supplementary Table 8** Average genomic prediction accuracies of Cu using single trait (ST) model (ST-GBLUP) and multi-trait (MT) models (MT-GBLUP and BMORS) under different cross-validation (CV) schemes. The highest prediction accuracy of MT models is underlined.

| CV scheme | Trait combinations | ST-GBLUP | MT-GBLUP | BMORS |
| --- | --- | --- | --- | --- |
| ST-CV1 | Cu | 0.21 |  |  |
| MT_CV2 | Cu+Mn |  | 0.08 | 0.15 |
|  | Cu+Fe |  | 0.09 | 0.14 |
|  | Cu+Zn |  | 0.07 | 0.16 |
|  | Cu+Cd |  | 0.09 | 0.16 |
|  | Cu+Mn+Fe |  | 0.08 | 0.16 |
|  | Cu+Mn+Zn |  | 0.09 | 0.17 |
|  | Cu+Mn+Cd |  | 0.07 | 0.15 |
|  | Cu+Fe+Zn |  | 0.08 | 0.18 |
|  | Cu+Fe+Cd |  | 0.06 | 0.15 |
|  | Cu+Zn+Cd |  | 0.09 | 0.19 |
|  | Cu+Mn+Fe+Zn |  | 0.08 | 0.16 |
|  | Cu+Mn+Fe+Cd |  | 0.06 | 0.14 |
|  | Cu+Mn+Zn+Cd |  | 0.06 | 0.16 |
|  | Cu+Fe+Zn+Cd |  | 0.07 | 0.17 |
|  | Cu+Mn+Fe+Zn+Cd |  | 0.06 | 0.18 |
| MT_CV3 | Cu+Mn |  | 0.07 | 0.12 |
|  | Cu+Fe |  | 0.07 | 0.13 |
|  | Cu+Zn |  | 0.08 | 0.10 |
|  | Cu+Cd |  | 0.08 | 0.11 |
|  | Cu+Mn+Fe |  | 0.07 | 0.12 |
|  | Cu+Mn+Zn |  | 0.08 | 0.10 |
|  | Cu+Mn+Cd |  | 0.09 | 0.12 |
|  | Cu+Fe+Zn |  | 0.08 | 0.10 |
|  | Cu+Fe+Cd |  | 0.08 | 0.11 |
|  | Cu+Zn+Cd |  | 0.06 | 0.12 |
|  | Cu+Mn+Fe+Zn |  | 0.08 | 0.10 |
|  | Cu+Mn+Fe+Cd |  | 0.09 | 0.13 |
|  | Cu+Mn+Zn+Cd |  | 0.06 | 0.11 |
|  | Cu+Fe+Zn+Cd |  | 0.06 | 0.08 |
|  | Cu+Mn+Fe+Zn+Cd |  | 0.06 | 0.10 |

**Supplementary Table 9** Average genomic prediction accuracies of Fe using single trait (ST) model (ST-GBLUP) and multi-trait (MT) models (MT-GBLUP and BMORS) under different cross-validation (CV) schemes. The highest prediction accuracy of MT models is underlined.

| CV scheme | Trait Combinations | ST-GBLUP | | MT-GBLUP | BMORS |  |
| --- | --- | --- | --- | --- | --- | --- |
| ST-CV1 | Fe | | 0.39 |  |  |  |
| MT_CV2 | Fe+Mn | |  | 0.19 | 0.27 |  |
|  | Fe+Cu | |  | 0.19 | 0.27 |  |
|  | Fe+Zn | |  | 0.19 | 0.27 |  |
|  | Fe+Cd | |  | 0.18 | 0.30 |  |
|  | Fe+Mn+Cu | |  | 0.17 | 0.26 |  |
|  | Fe+Mn+Zn | |  | 0.21 | 0.26 |  |
|  | Fe+Mn+Cd | |  | 0.20 | 0.30 |  |
|  | Fe+Cu+Zn | |  | 0.19 | 0.26 |  |
|  | Fe+Cu+Cd | |  | 0.20 | 0.31 |  |
|  | Fe+Zn+Cd | |  | 0.17 | 0.28 |  |
|  | Fe+Mn+Cu+Zn | |  | 0.20 | 0.27 |  |
|  | Fe+Mn+Cu+Cd | |  | 0.17 | 0.29 |  |
|  | Fe+Mn+Zn+Cd | |  | 0.22 | 0.31 |  |
|  | Fe+Cu+Zn+Cd | |  | 0.20 | 0.30 |  |
|  | Fe+Mn+Cu+Zn+Cd | |  | 0.21 | 0.32 |  |
| MT_CV3 | Fe+Mn |  | | 0.19 | 0.27 |  |
|  | Fe+Cu |  | | 0.20 | 0.26 |  |
|  | Fe+Zn |  | | 0.20 | 0.24 |  |
|  | Fe+Cd |  | | 0.19 | 0.27 |  |
|  | Fe+Mn+Cu |  | | 0.18 | 0.26 |  |
|  | Fe+Mn+Zn |  | | 0.20 | 0.23 |  |
|  | Fe+Mn+Cd |  | | 0.17 | 0.26 |  |
|  | Fe+Cu+Zn |  | | 0.17 | 0.23 |  |
|  | Fe+Cu+Cd |  | | 0.19 | 0.27 |  |
|  | Fe+Zn+Cd |  | | 0.20 | 0.25 |  |
|  | Fe+Mn+Cu+Zn |  | | 0.18 | 0.24 |  |
|  | Fe+Mn+Cu+Cd |  | | 0.19 | 0.25 |  |
|  | Fe+Mn+Zn+Cd |  | | 0.18 | 0.23 |  |
|  | Fe+Cu+Zn+Cd |  | | 0.17 | 0.22 |  |
|  | Fe+Mn+Cu+Zn+Cd |  | | 0.20 | 0.25 |  |

**Supplementary Table 10** Average genomic prediction accuracies of Cd using single trait (ST) model (ST-GBLUP) and multi-trait (MT) models (MT-GBLUP and BMORS) under different cross-validation (CV) schemes. The highest prediction accuracy of MT models is underlined.

| CV scheme | Trait combinations | ST-GBLUP | MT-GBLUP | BMORS |
| --- | --- | --- | --- | --- |
| ST-CV1 | Cd | 0.52 |  |  |
| MT_CV2 | Cd+Mn |  | 0.40 | 0.37 |
|  | Cd+Fe |  | 0.40 | 0.39 |
|  | Cd+Cu |  | 0.40 | 0.36 |
|  | Cd+Zn |  | 0.39 | 0.38 |
|  | Cd+Mn+Fe |  | 0.39 | 0.38 |
|  | Cd+Mn+Cu |  | 0.39 | 0.37 |
|  | Cd+Mn+Zn |  | 0.40 | 0.38 |
|  | Cd+Fe+Cu |  | 0.41 | 0.39 |
|  | Cd+Fe+Zn |  | 0.39 | 0.40 |
|  | Cd+Cu+Zn |  | 0.41 | 0.38 |
|  | Cd+Mn+Fe+Cu |  | 0.39 | 0.39 |
|  | Cd+Mn+Fe+Zn |  | 0.38 | 0.39 |
|  | Cd+Mn+Cu+Zn |  | 0.37 | 0.37 |
|  | Cd+Fe+Cu+Zn |  | 0.37 | 0.39 |
|  | Cd+Mn+Fe+Cu+Zn |  | 0.40 | 0.37 |
| MT_CV3 | Cd+Mn |  | 0.39 | 0.36 |
|  | Cd+Fe |  | 0.39 | 0.36 |
|  | Cd+Cu |  | 0.40 | 0.36 |
|  | Cd+Zn |  | 0.40 | 0.35 |
|  | Cd+Mn+Fe |  | 0.37 | 0.37 |
|  | Cd+Mn+Cu |  | 0.40 | 0.36 |
|  | Cd+Mn+Zn |  | 0.37 | 0.36 |
|  | Cd+Fe+Cu |  | 0.40 | 0.35 |
|  | Cd+Fe+Zn |  | 0.40 | 0.36 |
|  | Cd+Cu+Zn |  | 0.38 | 0.36 |
|  | Cd+Mn+Fe+Cu |  | 0.39 | 0.34 |
|  | Cd+Mn+Fe+Zn |  | 0.40 | 0.37 |
|  | Cd+Mn+Cu+Zn |  | 0.42 | 0.38 |
|  | Cd+Fe+Cu+Zn |  | 0.40 | 0.37 |
|  | Cd+Mn+Fe+Cu+Zn |  | 0.39 | 0.37 |

**Supplementary Table 11** Average genomic prediction accuracies of Mn using single trait (ST) model (ST-GBLUP) and multi-trait (MT) models (MT-GBLUP and BMORS) under different cross-validation (CV) schemes. The highest prediction accuracy of MT models is underlined.

| CV scheme | Trait combinations | ST-GBLUP | MT-GBLUP | BMORS |
| --- | --- | --- | --- | --- |
| ST-CV1 | Mn | 0.37 |  |  |
| MT_CV2 | Mn+Fe |  | 0.24 | 0.19 |
|  | Mn+Cu |  | 0.25 | 0.21 |
|  | Mn+Zn |  | 0.26 | 0.22 |
|  | Mn+Cd |  | 0.25 | 0.20 |
|  | Mn+Fe+Cu |  | 0.26 | 0.18 |
|  | Mn+Fe+Zn |  | 0.24 | 0.21 |
|  | Mn+Fe+Cd |  | 0.25 | 0.20 |
|  | Mn+Cu+Zn |  | 0.26 | 0.18 |
|  | Mn+Cu+Cd |  | 0.23 | 0.21 |
|  | Mn+Zn+Cd |  | 0.25 | 0.20 |
|  | Mn+Fe+Cu+Zn |  | 0.24 | 0.18 |
|  | Mn+Fe+Cu+Cd |  | 0.25 | 0.19 |
|  | Mn+Fe+Zn+Cd |  | 0.24 | 0.21 |
|  | Mn+Cu+Zn+Cd |  | 0.25 | 0.18 |
|  | Mn+Fe+Cu+Zn+Cd |  | 0.26 | 0.19 |
| MT_CV3 | Mn+Fe |  | 0.24 | 0.19 |
|  | Mn+Cu |  | 0.24 | 0.19 |
|  | Mn+Zn |  | 0.26 | 0.16 |
|  | Mn+Cd |  | 0.26 | 0.20 |
|  | Mn+Fe+Cu |  | 0.24 | 0.19 |
|  | Mn+Fe+Zn |  | 0.26 | 0.16 |
|  | Mn+Fe+Cd |  | 0.25 | 0.20 |
|  | Mn+Cu+Zn |  | 0.25 | 0.17 |
|  | Mn+Cu+Cd |  | 0.25 | 0.19 |
|  | Mn+Zn+Cd |  | 0.25 | 0.17 |
|  | Mn+Fe+Cu+Zn |  | 0.26 | 0.17 |
|  | Mn+Fe+Cu+Cd |  | 0.25 | 0.19 |
|  | Mn+Fe+Zn+Cd |  | 0.25 | 0.16 |
|  | Mn+Cu+Zn+Cd |  | 0.25 | 0.17 |
|  | Mn+Fe+Cu+Zn+Cd |  | 0.24 | 0.18 |
